# Free Vibration Analysis of Thin Functionally Graded Plate Bands with Microstructure as a Function of Material Inhomogeneity Distribution and Boundary Conditions

**DOI:** 10.3390/ma18194629

**Published:** 2025-10-07

**Authors:** Jarosław Jędrysiak, Magda Kaźmierczak-Sobińska

**Affiliations:** Department of Structural Mechanics, Łódź University of Technology, al. Politechniki 6, 90-924 Łódź, Poland

**Keywords:** functionally graded plates, tolerance-periodic microstructure, effect of microstructure, tolerance modelling, free vibrations

## Abstract

An analysis of free vibrations for thin functionally graded plate bands is presented in this work. On the microlevel these plate bands have a tolerance-periodic microstructure in planes parallel to the mid-plane. Partial differential equations with tolerance-periodic, highly oscillating, non-continuous coefficients describe the vibrations of such plates. Here, the influence of microstructure inhomogeneity is shown on free vibration frequencies of these plate bands with different boundary conditions. This analysis was carried out within the framework of two models of these plates. The models are represented by equations with smooth, slowly varying coefficients. One of these models, called the tolerance model, takes into account the effect of the microstructure size. Hence, it leads not only to formulas of fundamental lower-order vibration frequencies, but also to formulas of higher-order vibration frequencies, which are related to the microstructure. The analyses of free vibration frequencies for thin functionally graded plate bands with different boundary conditions are presented. The formulas of frequencies are obtained using the Ritz method. A comparison of some calculated results to the results obtained by the FEM is also shown.

## 1. Introduction

### 1.1. Subject of Analysis and Aim of the Work

**Figure 1:**
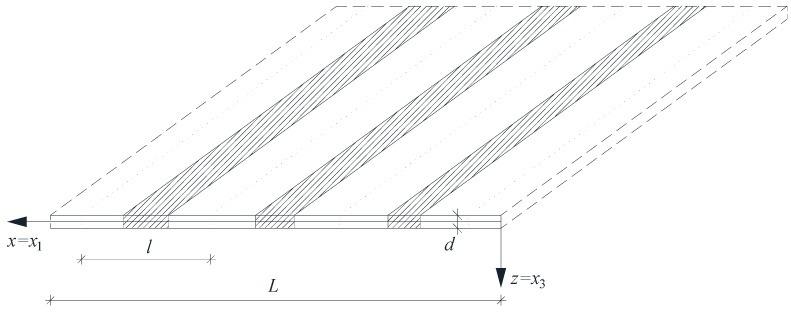
A fragment of a thin tolerance-periodic plate band.

Microheterogeneous structures, including beams, plates, and shells, are typically distinguished by their adequate rigidity and minimal weight. Consequently, they are frequently employed as a constituent element in a variety of structural systems within civil engineering, marine engineering, mechanical engineering, aerospace engineering, and railway engineering. This paper focuses on functionally graded (FG) plate band structures with a tolerance-periodic (non-periodic) microstructure. These plate structures are composed of numerous small elements, which may be called cells (see Figure 1).

The main aim of this work is to consider free vibrations of tolerance-periodic plate bands with different boundary conditions using *the tolerance* and *asymptotic models of dynamic problems for thin elastic tolerance-periodic (functionally graded with microstructure) plates*, cf. [1,2,3]. Moreover, the aim is also to investigate the influence of the various material tolerance-periodic cell structures and the various forms of edge support on free vibration frequencies. Applying the Ritz method, the formulae of these frequencies are derived. Some results are compared and justified by the finite element method. This work, despite its similar subject matter to that presented in [4], constitutes an interesting and valuable extension of it, as it deals with plate bands of functionally graded properties with microstructure. The analysed computational examples take into account different distributions of material properties in the plate plane, defined by different functions.

### 1.2. Literature Review

Because the microstructure of these plate bands is tolerance-periodic (non-periodic) along their span, their macrostructure can be regarded as functionally graded along this direction (see Suresh and Mortensen [1], Woźniak et al. [2]). These plate structures frequently find applications across numerous fields of modern engineering. The dynamic problems for the thin FG plates under consideration are governed by partial differentiation equations with highly oscillating, tolerance-periodic, and non-continuous coefficients. This form of the governing equations is, however, not convenient for their further analytical or numerical treatment. Consequently, various approximate averaging methods are typically introduced in the literature.

Some averaging techniques are devised for the analysis of periodic structures. Such methods are frequently used in the study of microstructured, functionally graded media, including plate structures (see [1,2]). Averaged models with effective (or homogeneous) properties—for instance plate stiffness or mass density—are then formulated within many of these methods. Among them, models based on *asymptotic homogenisation* [5] deserve special mention. In these models, plate behaviour is described by governing equations for a homogeneous plate with constant effective stiffness and mass density (see [6,7,8]). The asymptotic homogenisation procedure involves solving boundary value problems for the representative periodic cell to compute these effective properties. This procedure typically involves retaining only the first approximation, while microstructure size effects are neglected in the resulting macroscopic equations.

Other modelling approaches for composite media are successfully formulated and implemented in related problems. Some of these methods are referenced in this paper—predominantly in relation to plate and shell structures. Homogenisation with *microlocal parameters* is used to investigate periodic plate structures [9], microperiodic composite half-planes with slant lamination [10], or a semi-infinite homogeneous medium with a multilayer coating assembly of periodic cells [11]. The free vibration frequencies of thick square panels made of orthotropic or hexagonal materials are considered in [12]. The stability of multicell thin-walled columns is studied in [13]. Two approximate methods—orthogonalisation and finite difference are used in [14] to investigate dynamics for sandwich annular plate structures with a viscoelastic core. Buckling and post-buckling problems of shells of revolution with non-classical geometry are shown in [15] employing analytical–numerical models. Furthermore, analytical–numerical approaches are used in [16] to consider the buckling of sandwich polyethylene plates under a magnetic field. Paper [17] shows that computer simulations enable the study of the effective properties and dynamic response of a sandwich panel with an auxetic core. An analytical–numerical approach is applied in [18] to investigate dynamic problems related to fluid flow in plate structures with different Poisson’s ratios. In [19] a comparison of the blast resistance for auxetic and non-auxetic sandwich plates is made using the finite element method. A certain computational approach based on polygonal meshes to describe free vibrations, buckling, and dynamic instability problems of the sandwich plate structures with an auxetic honeycomb core is proposed in [20]. Sandwich plates with an auxetic, anti-tetrachiral core are analysed under steady-state harmonic base motion in [21] with the aid of the finite element method. The results for auxetic sandwich plates are then contrasted with those for standard honeycomb-cored structures. Orthogonalisation and the finite difference method are used for composite annular plate structures with auxetic properties under static stability [22] or dynamic stability [23]. The exact strong form of the equations for Timoshenko–Ehrenfest beams with geometric nonlinearity is derived in [24], and their weak form is subsequently obtained by the finite element method. Natural frequencies, mode shapes, and nonlinear free vibrations are computed. In [25], the dynamic stability of a Mindlin–Reissner plate is investigated employing a variational approach to construct its stiffness matrix alongside Floquet theory and a first-order approximation.

Numerous works present a range of theoretical and numerical results for various problems related to functionally graded structures. *Higher-order theories* for thermomechanical problems in functionally graded, microstructured composite materials are developed in [26,27,28,29]. The boundary element method is successfully used to conduct thermal analysis of composite materials with fibres in [30]. Furthermore, a specialised implementation of the finite element method for functionally graded materials is considered in [31]. The stability of cylindrical shells with functionally graded structures is investigated in [32] and shown to be in accord with Donnell-type dynamic stability equations. Meshless methods are applied in [33] to compute the natural frequencies of composite plate structures, while in [34] the dynamic response of sandwich beams with a functionally graded core is studied. In [35] vibrations of functionally graded plates are analysed using higher-order plate theories and a collocation method. A GDQ solution is applied in [36] for free vibrations of shells. Thermomechanical problems of a functionally graded plate and shell structures are considered in [37,38] using higher-order shear deformation plate theories. In [39,40,41] the static behaviour of doubly curved, functionally graded shells is investigated. The thermal buckling of annular functionally graded plates is considered in [42], applying the non-classical FG plate model, based on the modified couple stress theory, where size effects related to couple stress are taken into account. In [43] free vibrations of functionally graded thick plates are investigated with consideration of both normal and shear deformations. Paper [44] shows an application of higher-order plate theory to vibrations of rectangular FG plate structures. A nonlinear analysis employing shear deformation plate theory is presented in [45]. The chaos phenomenon for a rectangular FG plate is investigated in [46]. A strong-form formulation employing the GDQ technique alongside the finite element method for multilayered plate structures is presented in [47], while [48] shows a strong-form isogeometric analysis for composite multilayer plate structures. The differential quadrature method and a layer-wise plate theory are used to compute vibrations of plate structures in [49]. A new low-order shell element for composite shell structures with functional gradation is introduced in [50]. The differential quadrature method is successfully used in many related problems—for instance, to compute the natural frequencies of sandwich shells [51] or to assess their dynamic stability [52]. Furthermore, sinusoidal shear deformation plate theory is applied to investigate bending of piezoelectric functionally graded plate structures resting on a foundation and free vibrations of functionally graded composite polymer nanoplates in [53,54]. The classical laminate plate theory is employed to propose a semi-analytical method for analysing bending, post-buckling, and dynamic problems in functionally graded thin plate structures (see [55]). Columns with open or closed cross-sections made of laminate plate structures are analysed (see [56,57,58]) using the classical laminate plate theory. Free vibrations of sandwich functionally graded plates under thermal loadings are considered applying a 3D finite element formulation in [59]. Transient behaviour under in-plane displacements and temperature effects is investigated in [60], employing a new semi-analytical algorithm. In [61] an analytical method using complex variables is applied to compute forces and moments in infinite, symmetric, functionally graded plate structures with a hole. A four-node finite element based on a simple high-order shear deformation theory is presented in [62] to analyse buckling problems of functionally graded rectangular plates under mechanical and thermal loading. A new analytical model for sandwich plate structures is proposed in [63] and subsequently generalised to account for a thickness-wise variation in their mechanical properties. The model is developed within a nonlinear theory in which a straight normal to the plate’s mid-plane deforms. A bending of a clamped sandwich beam with a functionally graded core under a uniformly distributed load is considered in [64] using a nonlinear shear deformation theory with the classical shear stress formula for beams. In [65] static temperature distribution in a three-layered, annular plate with heterogeneous facings made of material with radially variable parameters and with a thicker foam core is analysed by applying the finite difference method. An analytical model for the elastic buckling of a sandwich plate with an individually graded core, employing the nonlinear shear deformation theory of a straight normal, is shown in [66]. The vibrations of porous, functionally graded material plate resting on a Winkler foundation are investigated in [67] using first-order shear deformation plate theory alongside the variational Galerkin–Vlasov method. Vibrations of FG plate structures are considered in [68] applying the dynamic stiffness method. Paper [69] introduces a unified solution for the transient state vibrations of porous FG plate structures employing a combination of the Jacobi–Ritz method and a higher-order shear deformation plate theory. In [70] a unified size-dependent shear deformation theory based on consistent couple stress theory is proposed to consider the dynamics of a functionally graded magneto-electro-elastic microplate under biaxial compression, magnetic and electric potentials, and uniform temperature changes. Using the generalised differential quadrature method, vibrations of nanocomposite FG shells are analysed in [71,72,73]. In [74] a highly accurate and convenient analytical model for statical mechanics of pressurised FG annular structures with arbitrary stiffness variation in the radial direction is shown. In [75] an investigation into low-frequency vibrations of a thin-walled FG cylinder employing a plane strain framework is performed. Subsequently, an asymptotic analysis of the dynamic relationships in elasticity is carried out across the cylinder’s cross-section, yielding a consistent approximate equation of motion for its mid-plane.

It should be marked, however, that equations of models derived within the framework of the methods discussed above typically neglect microstructure size effects—a consideration that can be significant in the context of vibrations in microstructured media. As Brillouin [76] observes, there are relationships between macro- and microvibrations—the former pertaining to the macrostructure and the latter to its microstructure. To account for this, specialised methods are implemented in numerous studies. This includes periodic structures, as documented in many subsequent works. In [77] a spectral element method is used to analyse the characteristics of vibration band gaps in Mindlin’s periodic plate structures. In [78,79] a centre-difference method is employed to study band gaps in periodic thin plate structures with and without damping. In [80] the differential quadrature element method is used to investigate the flexural wave band gaps in composite periodic plate structures.

The *tolerance method* (also called *tolerance modelling*) offers a powerful alternative for analysing different mechanical problems in microstructured media—whether periodic or non-periodic. For further details, see the monograph by Woźniak and Wierzbicki [81] or Woźniak et al. [2,82]. This method applies to a range of problems governed by partial differential equations with functional highly oscillating, non-continuous coefficients. In its framework, the exact governing equations are replaced by averaged ones with constant or slowly varying coefficients. Some of these coefficients are explicitly dependent on the microstructure size.

This procedure makes it possible to investigate a range of dynamical, stability, and thermo-elastic problems related to periodic structures, as discussed in numerous articles. Some noteworthy examples include the analysis of fluid-saturated periodic grounds [83] or the vibrations of periodic plane structures [84]. Applications to dynamics of periodic medium-thickness plate structures are presented in [85], but for in-plane periodic thin plate structures with thicknesses less than the periodic cell dimension, see [86]. The vibrations of wavy periodic plate structures are studied in [87]. The dynamics of thin periodic plate structures with stiffeners are investigated in [88]. The vibrations of periodic thin plate structures with a microstructure size comparable to plate thickness are considered in [89]. Applications to various thermomechanical problems, including stability and vibrations of thin cylindrical shells with two-directional or one-directional microperiodicity, are shown in [90,91,92]. Periodic plate structures with medium deflections are considered in [93], while [94] studied the geometric nonlinearity in periodic beams. A specialised tolerance model for vibrations in periodic, sandwich plate structures is presented in [95], with a comparison of several dynamic models, and in [96]. The tolerance method is also applied to analyse a problem of heat transfer for periodic laminates with probabilistic distribution of material properties in [97]. The tolerance method alongside the finite difference method is used to investigate the heat conduction process in biperiodic composite materials [98,99]. A certain generalised tolerance model of dynamics and stability for visco-elastic periodic beams on a periodic damping foundation is proposed in [100]. The multiscale stress distribution in composite periodic thin plate structures is considered in [101].

The tolerance method is also successfully applied to the modelling of non-periodic, microstructured media. Thermo-elastic problems in transversally graded laminates are investigated in [102]. The vibrations of longitudinally graded plate structures are studied in [103,104], while their stability is examined in [105]. In [106,107] the authors use tolerance models for the dynamic analysis of thin-walled structures with dense stiffening elements. Heat transfer in cylindrical composite conductors with non-uniform distribution of constituents is investigated in [108,109]. A further study of conductive properties under Robin boundary conditions for multilayer structures with grading in material properties is presented in [110]. A certain generalisation of existing tolerance models of heat conduction in two-component stepwise functionally graded materials is considered in [111]. The vibrations of thin, transversally graded plate structures with a thickness less than the microstructure size are shown in [2,3]. The free vibrations of thin, functionally graded plate structures with a microstructure size on the order of plate thickness are analysed in [112], but for medium thickness plates, see [113]. The dynamic problems of functionally graded microstructured thin shells are considered in [114,115,116], and their stability is considered in [117]. However, it is worth noting that these works do not encompass all the problems previously discussed in the literature related to tolerance modelling for microstructured media; therefore, the state of knowledge in this area cannot be considered exhaustive.

## 2. Foundations

### 2.1. Preliminaries

Notations for coordinates are introduced: *x = x*_1_, *z = x*_3_, *x* ∈ [0, *L*], *z* ∈ [*−d*/2, *d*/2], with *d* as a constant plate thickness, and *L* as a span of the plate band. The considerations are assumed to be independent of the *x_2_*-coordinate. The plate band is determined by an interval Π, Π = (0, *L*). Moreover, what is called “the basic cell” Δ ≡ [*−l*/2, *l*/2] × {0} is introduced as an interval in Π, with *l* being the cell length, which satisfies conditions *d* << *l* << *L*. It is assumed that the plate band consists of two elastic, isotropic materials, perfectly bonded across interfaces. Their properties are denoted as Poisson’s ratios *v*′, *v*″, Young’s moduli *E*′, *E*″, and mass densities *ρ*′, *ρ*″. In the next considerations, it is assumed that *v*′ = *v*″, and *E*′ ≠ *E*″ or *ρ*′ ≠ *ρ*″. Indices *A*, *B*, … run over 1, …, *N* and summation convention holds for them. Let ∂ denote the first derivative with respect to *x*, and ∂*^n^*—the derivative of the *n*-th order; however, the overdot and the overdots denote derivatives with respect to the time coordinate.

The plate band properties are described by tolerance-periodic functions in *x*: the bending stiffness *b*, the mass density μ, the rotational inertia *j*, given by:

(1) $$b(x)=\frac{d^{3}}{12(1-\nu^{2})}E(x),\;\;\;\;\mu (x)=d\rho (x),\;\;\;\;j(x)=\frac{d^{3}}{12}\rho (x).$$

Let *w*(*x*,*t*) denote the deflection of the plate band (*x* ∈ Π, *t* ∈ (*t*_0_, *t*_1_)). Using the well-known assumptions of the Kirchhoff-type plate theory, free vibrations of thin functionally graded plate band are described by the fourth-order partial differential equation in the following form:

(2) $$\partial^{2}(b\partial^{2}w)+\mu \ddot{w}-j\partial^{2}\ddot{w}=0,$$

which has discontinuous and highly oscillating, tolerance-periodic functional coefficients. Hence, using various mathematical approaches this equation is often replaced by an averaged equation, with smooth coefficients.

In order to maintain the possibility to analyse the effect of microstructure size in this study, *a tolerance modelling method* is employed.

### 2.2. The Tolerance Modelling

*The tolerance modelling method* includes some introductory concepts, as were defined in a general form in the book [2] and also for various media in a series of papers, e.g., for beams in [94,100], and for non-periodic plates in [3,112]. Thus, they are only listed here, i.e., the averaging operator <·> (which is presented below for plate bands), the tolerance parameter, the tolerance-periodic function *TP*(Δ), the highly oscillating function *HO*(Δ), the fluctuation shape function *FS*(Δ), and the slowly varying function *SV*(Δ).

Let us introduce a cell at *x* ∈ Π_Δ_: Δ(*x*) = *x* + Δ, Π_Δ_ = {*x*∈Π: Δ(*x*) ⊂ Π}. For plate bands *the averaging operator* is formulated as

(3) $$<f>(x)={\left(l\right)}^{-1}\int_{\Delta (x)}f(\xi )d\xi ,\;x\in \Pi_{\Delta },\;\xi \in \Delta (x),$$

with *f* being an integrable function. If function *f* is tolerance-periodic in *x*, its averaged value from (3) is a slowly varying function.

Let us also recall another important concept, that of *the fluctuating shape function*, g∈FS(Δ), which is continuous together with gradient ∂^1^*g* and with a piecewise continuous and bounded gradient ∂^2^*g*. It depends on the microstructure parameter *l*, and satisfies the following conditions:

(4) $$\begin{array}{l} (i)\;\partial^{k}g\in O(l^{\alpha -k})\;\mathrm{for}\;k=0,1,\ldots ,\alpha ,\;\alpha =2,\;\partial^{0}g\equiv g,\\ (ii)\;<g>(x)\approx 0\forall x\in \Pi_{\Delta }. \end{array}$$

Condition (*ii*) may be replaced by <μ*g* > (*x*) ≈ 0 for every *x* ∈ Π_Δ_, with μ > 0 as a certain tolerance-periodic function.

The tolerance modelling is based on some fundamental assumptions, which are formulated in the book [2] in their general form. Their proper formulation for thin microstructured plates can be found, e.g., in [2,3,112]. Below, these assumptions are presented for thin tolerance-periodic plate bands.

*The micro–macro decomposition* assumes that the deflection can be decomposed as:

(5) $$w(x,t)=W(x,t)+h^{A}(x)Q^{A}(x,t),\;\;A=1,\ldots ,N,$$

using the basic unknowns: *the macrodeflection W*(·,*t*), *the fluctuation amplitudes Q^A^*(·,*t*), $W(\cdot ,t),\;Q^{A}(\cdot ,t)\in SV(\Delta )$, and the known fluctuation shape functions $h^{A}(\cdot )\in FS(\Delta )$. Fluctuation shape functions can be solutions to eigenvalue problems posed on the basic cell. However, they can usually be assumed in an approximate form as trigonometric functions [3], or saw-type functions [113].

*The tolerance averaging approximation*, on the other hand, assumes that terms *O*(δ) are negligibly small in the modelling procedure, and can be neglected in the formulas:

(6) $$\begin{array}{l} \begin{array}{ll} \left(i\right) & <\varphi >(x)=\;<\bar{\varphi }>(x)+O(\delta ),\\ \left(ii\right) & <\varphi F>(x)=\;<\varphi >(x)F(x)+O(\delta ),\\ \left(iii\right) & <\varphi \partial (gF)>(x)=\;<\varphi \partial g>(x)F(x)+O(\delta ), \end{array}\\ x\in \Pi ;\;0<\delta <<1;\;\varphi \in TP(\Delta ),\;F\in SV(\Delta ),\;g\in FS(\Delta ). \end{array}$$

Tolerance modelling is predicated on a foundation of introductory concepts and basic assumptions (5) and (6).

The modelling procedure itself can be carried out in a number of ways. Firstly, the virtual work principle, as outlined in [4], can be employed. Secondly, an averaged Lagrangian, as described in [112], can be utilised.

Alternatively, as in [100], a residual field *r*(·), defined as the left-hand side of Equation (2), can be formulated after inserting a micro–macro decomposition:

(7) $$\begin{array}{l} r(\cdot )=\partial^{2}[b\partial^{2}(W(x,t)+h^{A}(x)Q^{A}(x,t))]+\\ +\mu [\ddot{W}(x,t)+h^{A}(x){\ddot{Q}}^{A}(x,t)]-j\partial^{2}[\ddot{W}(x,t)+h^{A}(x){\ddot{Q}}^{A}(x,t)]. \end{array}$$

This field can then be restricted by introducing an additional assumption of *the residual orthogonality condition*:

(8) $$<r>(x,t)=0,\;\;<rh^{B}>(x,t)=0.$$

All the above-mentioned ways of carrying out the tolerance modelling procedure lead to the same averaged governing equations of plates or beams with microstructure.

## 3. Governing Equations of Tolerance-Periodic Plate Bands

### 3.1. Tolerance Model Equations

Introduce denotations:

(9) $$\begin{array}{lll} B\equiv \;<b>,\;\; & B^{A}\equiv \;<b\partial^{2}h^{A}>,\;\; & B^{AB}\equiv \;<b\partial^{2}h^{A}\partial^{2}h^{B}>,\\ m\equiv \;<\mu >,\;\; & m^{A}\equiv l^{-2}<\mu h^{A}>,\;\; & m^{AB}\equiv l^{-4}<\mu h^{A}h^{B}>,\;\;\\ \vartheta \equiv \;<j>,\;\; & \vartheta^{A}\equiv l^{-1}<j\partial h^{A}>,\;\; & \vartheta^{AB}\equiv l^{-2}<j\partial h^{A}\partial h^{B}>. \end{array}$$

Using the last of the abovementioned tolerance modelling procedures, the system of equations for the macrodeflection *W* and the fluctuation amplitudes of the deflection *Q^A^* is derived (here, in the form for tolerance-periodic plate bands):

(10) $$\begin{array}{l} \partial^{2}(B\partial^{2}W+B^{A}Q^{A})+m\ddot{W}+l^{2}m^{A}{\ddot{Q}}^{A}-\vartheta \partial^{2}\ddot{W}-l\vartheta^{A}\partial {\ddot{Q}}^{A}=0,\\ B^{A}\partial^{2}W+B^{AB}Q^{B}+l^{2}m^{A}\ddot{W}+l\vartheta^{A}\partial \ddot{W}+l^{2}(l^{2}m^{AB}+\vartheta^{AB}){\ddot{Q}}^{B}=0. \end{array}$$

Equation (10) constitutes *the tolerance model of free vibrations of thin elastic tolerance-periodic plate bands*, with slowly varying functional coefficients. These model equations, using terms with the microstructure parameter *l*, allow the effect of the microstructure size on the plate band’s free vibrations to be taken into account. Moreover, it should be noted that the basic unknowns of (10) have to be slowly varying functions in *x*, $W(\cdot ,t),\;Q^{A}(\cdot ,t)\in SV(\Delta )$. For these equations boundary conditions only for the macrodeflection *W* have to be defined.

### 3.2. Asymptotic Model Equations

In order to evaluate the results obtained with the tolerance model, a model that does not take into account the influence of microstructure size, i.e., an asymptotic model, will also be introduced. Its equations can be obtained by carrying out a formal asymptotic procedure, e.g., [82,112,113] or by simply deriving directly from Equation (10) by omitting components of order *O*(*l^n^*), *n* = 1, 2, ….

Using such transformations, the equations of *the asymptotic model* of free vibration for plate bands under consideration are obtained:

(11) $$\begin{array}{l} \partial^{2}(B\partial^{2}W+B^{A}Q^{A})+m\ddot{W}-\vartheta \partial^{2}\ddot{W}=0,\\ B^{A}\partial^{2}W+B^{AB}Q^{B}=0, \end{array}$$

with all slowly varying functional coefficients.

Therefore, *the asymptotic model of free vibrations of thin elastic tolerance-periodic plate bands* is defined by Equation (11) with boundary conditions also formulated only for the macrodeflection *W*.

## 4. Free Vibration Analysis for Tolerance-Periodic Plate Bands with Different Support Conditions

### 4.1. Introduction

The two plate band materials are characterised by constant Poisson’s ratios ν′ = ν″ = ν, the variable Young’s moduli *E*′, *E*″, and mass densities ρ′, ρ″. These materials are tolerance-periodically distributed along the *x*-axis and also perfectly bonded on interfaces.

**Figure 2:**
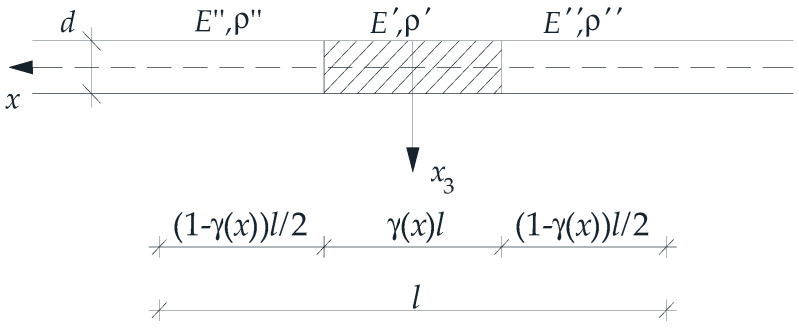
Tolerance-periodic plate band cell.

It is assumed that the properties of the plate band under consideration are described as follows:

(12) $$E(\cdot ,y),\;\rho (\cdot ,y)=\left\{\begin{array}{l} E^{\prime },\;\rho^{\prime }\;\mathrm{for}\;y\in ((1-\gamma (x))l/2,(1+\gamma (x))l/2),\\ E^{\prime \prime },\;\rho^{\prime \prime }\;\mathrm{for}\;y\in [0,(1-\gamma (x))l/2]\cup [(1+\gamma (x))l/2,l], \end{array}\right.$$

where γ(*x*) is *the distribution function of the material properties* (Figure 2); *y* ∈ Δ(*x*). Under the above assumptions, the influence of the tolerance-periodic material structure on the free vibrations is considered by taking the following parameter values: *E*″/*E*′∈[0, 1], ν″ = ν′ = ν = 0.3, ρ″/ρ′∈[0, 1], *h*/*l*∈(0; 0.1].

The impact of the above parameters is considered using the example of the first vibration frequencies, only—lower and higher (for the tolerance model).

Furthermore, only one fluctuation shape function *h*(*x*) = *h*^1^(*x*), *A* = *N* = 1, is assumed. Denote *Q* ≡ *Q*^1^. Then micro–macro decomposition (5) of the deflection *w*(*x*,*t*) of the plate band takes the form of

(13) w(x,t)=W(x,t)+h(x)Q(x,t),

where W(⋅,t), Q(⋅,t)∈SV(Δ) for every $t\in (t_{0},t_{1})$, h(⋅)∈FS(Δ).

For a given cell, as illustrated in Figure 2, a periodic approximation of the fluctuation shape function *h*(*x*) is employed, of the following form:

(14) $$\tilde{h}(x,y)=l^{2}[\cos(2\pi y/l)+c(x)],\;\;y\in \Delta (x),\;\;x\in \bar{\Pi },$$

where *c*(*x*) is determined by the condition $<\mu \tilde{h}>\;=0$:

(15) $$c=c(x)=\frac{\sin[\pi \tilde{\gamma }(x)](\rho^{\prime }-\rho^{\prime \prime })}{\pi \{\rho^{\prime }\tilde{\gamma }(x)+\rho^{\prime \prime }[1-\tilde{\gamma }(x)]\}},$$

with $\tilde{\gamma }(x)$ as the periodic approximation of the distribution function of material properties.

The quantity *c*(*x*) is a slowly varying function of the argument *x*. By determining the derivatives of the fluctuating shape function (performing a differentiation within the cell, relative to *y* ∈ Δ(*x*)), the quantity *c*(*x*) can be treated as a constant, obtaining:

(16) $$\partial \tilde{h}(y)=-2\pi l\sin(2\pi y/l),\;\;\;\partial^{2}\tilde{h}(y)=-4\pi^{2}\cos(2\pi y/l).$$

For one fluctuation shape function assumed as (14), denotations (9) can be written as:

(17) $$\begin{array}{lll} B\equiv \;<b>,\;\; & B^{1}\equiv \;<b\partial^{2}h>,\;\; & B^{11}\equiv \;<b\partial^{2}h\partial^{2}h>,\\ m\equiv \;<\mu >,\;\; & m^{1}\equiv l^{-2}<\mu h>,\;\; & m^{11}\equiv l^{-4}<\mu hh>,\\ \vartheta \equiv \;<j>,\;\; & \vartheta^{1}\equiv l^{-1}<j\partial h>,\;\; & \vartheta^{11}\equiv l^{-2}<j\partial h\partial h>; \end{array}$$

and

(18) $$m^{1}\equiv l^{-2}<\mu h>\;=0,\;\;\vartheta^{1}\equiv l^{-1}<j\partial h>\;=0.$$

Thus, Equation (10) of the tolerance model can be represented as:

(19) $$\begin{array}{l} \partial^{2}(B\partial^{2}W+B^{1}Q)+m\ddot{W}-\vartheta \partial^{2}\ddot{W}=0,\\ B^{1}\partial^{2}W+B^{11}Q+l^{2}(l^{2}m^{11}+\vartheta^{11})\ddot{Q}=0, \end{array}$$

After simple transformations from the second formula in Equation (11), the fluctuation amplitude *Q* can be determined. Then, this formula is substituted for the function *Q* in the first formula in these equations, and after rearranging the asymptotic model equation, it can be written as a single equation only for the macrodeflection *W*:

(20) $$\partial^{2}\{[B-{\left(B^{1}\right)}^{2}/B^{11}]\partial^{2}W\}+m\ddot{W}-\vartheta \partial^{2}\ddot{W}=0.$$

Despite the simplification of the primary Equation (2) (with strongly oscillating, discontinuous functional coefficients), finding analytical solutions of the averaged equations presented above (19) or (20) (with continuous functional coefficients) is still very difficult or even impossible; so approximate methods are used for this purpose. In this paper, the Ritz method is used to write approximate formulae for the natural frequencies of the plates under consideration.

### 4.2. The Application of the Ritz Method

The Ritz method may be used to derive the free vibration frequency formulae for functionally graded plate bands with tolerance-periodic microstructure, which are different support conditions, cf. [3,4]. This method uses the concepts of maximum strain energy $U_{\max}$ and maximum kinetic energy $K_{\max}$, for which the relevant formulae must be formulated.

Assume solutions to Equation (20) and Equation (19) for the considered plate bands in the following form:

(21) $$W(x,t)=A_{W}\Phi (\alpha x)\cos(\omega t),\;\;Q(x,t)=A_{Q}\Psi (\alpha x)\cos(\omega t),$$

where α is a wave number, ω is a free vibration frequency, and *A_W_* and *A_Q_* are amplitudes. Functions Φ(·) and Ψ(·) are eigenfunctions for the macrodeflection and the fluctuation amplitude, respectively, which have to satisfy the given boundary conditions for *x* = 0, *L*. Also denote the first- and second-order derivatives of functions Φ(·) and Ψ(·) by:

(22) $$\begin{array}{l} \partial \Phi (\alpha x)\equiv \alpha \tilde{\Phi }(\alpha x),\;\;\partial \Psi (\alpha x)\equiv \alpha \tilde{\Psi }(\alpha x),\\ \partial \partial \Phi (\alpha x)\equiv \alpha^{2}\bar{\Phi }(\alpha x),\;\;\partial \partial \Psi (\alpha x)\equiv \alpha^{2}\bar{\Psi }(\alpha x). \end{array}$$

The following analysis will be carried out for four cases of boundary conditions:

- The simply supported plate band

(23) Φ(0)=∂∂Φ(0)=Φ(L)=∂∂Φ(L)=0;

- The plate band clamped on both edges

(24) Φ(0)=∂Φ(0)=Φ(L)=∂Φ(L)=0;

- The clamped–hinged plate band

(25) Φ(0)=∂Φ(0)=Φ(L)=∂∂Φ(L)=0;

- The cantilever plate band

(26) Φ(0)=∂Φ(0)=∂∂Φ(L)=∂∂∂Φ(L)=0.

The eigenfunctions Ψ(·) and Φ(·) from the solutions of (21) can be taken as a homogeneous plate band satisfying the proper boundary conditions (23)–(26). The notation of these solutions uses a combination of trigonometric–hyperbolic functions:

(27) $$\begin{array}{l} S(\alpha x)=\frac{1}{2}[\cosh(\alpha x)+\cos(\alpha x)],\;T(\alpha x)=\frac{1}{2}[\sinh(\alpha x)+\sin(\alpha x)],\\ U(\alpha x)=\frac{1}{2}[\cosh(\alpha x)-\cos(\alpha x)],\;V(\alpha x)=\frac{1}{2}[\sinh(\alpha x)-\sin(\alpha x)]. \end{array}$$

Therefore, the eigenfunctions Ψ(·) and Φ(·) take the form:

- The simply supported plate band

(28) Φ(αx)=Ψ(αx)=sin(αx);

- The plate band clamped on both edges

(29) $$\Phi (\alpha x)=\Psi (\alpha x)=U(\alpha x)-\frac{\cosh(\alpha L)-\cos(\alpha L)}{\sinh(\alpha L)-\sin(\alpha L)}V(\alpha x);$$

- The clamped–hinged plate band

(30) Φ(αx)=Ψ(αx)=U(αx)−coth(αL)V(αx);

- The cantilever plate band

(31) $$\Phi (\alpha x)=\Psi (\alpha x)=U(\alpha x)-\frac{\sinh(\alpha L)-\sin(\alpha L)}{\cosh(\alpha L)+\cos(\alpha L)}V(\alpha x).$$

It is now necessary to determine the formulae for the maximum strain energy $U_{\max}$ and maximum kinetic energy $K_{\max}$ for both the tolerance and asymptotic models within the Ritz method. The conditions of the Ritz method are then used:

(32) $$\frac{\partial (U_{\max}-K_{\max})}{\partial A_{W}}=0,\;\;\frac{\partial (U_{\max}-K_{\max})}{\partial A_{Q}}=0,$$

which allow the formulae for the free vibration frequencies to be determined.

After substituting into (17) the fluctuation shape function (14), the functions of material properties (12), and eigenfunctions Φ(·) and Ψ(·), denotations (17) can be written as:

(33) $$\begin{array}{l} \overset{⌣}{B}=\frac{d^{3}}{12(1-\nu^{2})}\int_{0}^{L}\{E^{\prime \prime }[1-\tilde{\gamma }(x)]+\tilde{\gamma }(x)E^{\prime }\}{\left[\bar{\Phi }(\alpha x)\right]}^{2}dx,\\ \bar{B}=\frac{\pi d^{3}}{3(1-\nu^{2})}(E^{\prime }-E^{\prime \prime })\int_{0}^{L}\sin(\pi \tilde{\gamma }(x))\bar{\Phi }(\alpha x)\Psi (\alpha x)dx,\\ \overset{⌢}{B}=\frac{{\left(\pi d\right)}^{3}}{3(1-\nu^{2})}\int_{0}^{L}\{(E^{\prime }-E^{\prime \prime })[2\pi \tilde{\gamma }(x)+\sin(2\pi \tilde{\gamma }(x))]+2\pi E^{\prime \prime }\}{\left[\Psi (\alpha x)\right]}^{2}dx,\\ \overset{⌣}{\mu }=d\int_{0}^{L}\{[1-\tilde{\gamma }(x)]\rho^{\prime \prime }+\tilde{\gamma }(x)\rho^{\prime }\}{\left[\Phi (\alpha x)\right]}^{2}dx,\\ \bar{\mu }=\frac{d}{4\pi }\int_{0}^{L}\{(\rho^{\prime }-\rho^{\prime \prime })[2\pi \tilde{\gamma }(x)+\sin(2\pi \tilde{\gamma }(x))]+2\pi \rho^{\prime \prime }\}{\left[\Psi (\alpha x)\right]}^{2}dx+\\ \;\;+\frac{d}{\pi }(\rho^{\prime }-\rho^{\prime \prime })\int_{0}^{L}c(x)[\pi c(x)\tilde{\gamma }(x)-2\sin(\pi \tilde{\gamma }(x))]{\left[\Psi (\alpha x)\right]}^{2}dx+\\ \;\;+d\rho^{\prime \prime }\int_{0}^{L}{\left[c(x)\right]}^{2}{\left[\Psi (\alpha x)\right]}^{2}dx,\\ \overset{⌣}{\vartheta }=\frac{d^{3}}{12}\int_{0}^{L}\{[1-\tilde{\gamma }(x)]\rho^{\prime \prime }+\tilde{\gamma }(x)\rho^{\prime }\}{\left[\tilde{\Phi }(\alpha x)\right]}^{2}dx,\\ \bar{\vartheta }=\frac{\pi d^{3}}{12}\int_{0}^{L}\{(\rho^{\prime }-\rho^{\prime \prime })[2\pi \tilde{\gamma }(x)-\sin(2\pi \tilde{\gamma }(x))]+2\pi \rho^{\prime \prime }\}{\left[\Psi (\alpha x)\right]}^{2}dx. \end{array}$$

Using (21) and the above notations (33), formulae of the maximal strain energy $U_{\max}$ and the maximal kinetic energy $K_{\max}$ for the plate band by the tolerance model take the form:

(34) $$U_{\max}^{TM}=\frac{1}{2}(\overset{⌣}{B}A_{W}^{2}\alpha^{4}+2\bar{B}A_{W}A_{Q}\alpha^{2}+\overset{⌢}{B}A_{Q}^{2}),\;\;K_{\max}^{TM}=\frac{1}{2}[(\overset{⌣}{\mu }+\overset{⌣}{\vartheta }\alpha^{2})A_{W}^{2}+l^{2}(\bar{\mu }l^{2}+\bar{\vartheta })A_{Q}^{2}]\omega^{2}.$$

After substituting formulae (34) into the conditions of the Ritz method (32), the following system of linear algebraic equations is obtained:

(35) $$\begin{array}{l} A_{W}[\overset{⌣}{B}\alpha^{4}-(\overset{⌣}{\mu }+\overset{⌣}{\vartheta }\alpha^{2})\omega^{2}]+A_{Q}\bar{B}\alpha^{2}=0,\\ A_{W}\bar{B}\alpha^{2}+A_{Q}[\overset{⌢}{B}-l^{2}(\bar{\mu }l^{2}+\bar{\vartheta })\omega^{2}]=0. \end{array}$$

Assigning the determinant of the system (35) to zero gives the following free vibration characteristic equation for a tolerance-periodic plate band according to the tolerance model:

(36) $$l^{2}(\bar{\mu }l^{2}+\bar{\vartheta })(\overset{⌣}{\mu }+\overset{⌣}{\vartheta }\alpha^{2})\omega^{4}-[\overset{⌣}{B}\alpha^{4}l^{2}(\bar{\mu }l^{2}+\bar{\vartheta })+\overset{⌢}{B}(\overset{⌣}{\mu }+\overset{⌣}{\vartheta }\alpha^{2})]\omega^{2}+\overset{⌣}{B}\overset{⌢}{B}\alpha^{4}-{\left(\bar{B}\alpha^{2}\right)}^{2}=0.$$

The solutions to Equation (36) can be written in the following form:

(37) $$\begin{array}{l} {\left(\omega_{-}\right)}^{2}=\frac{\alpha^{4}l^{2}(\bar{\mu }l^{2}+\bar{\vartheta })\overset{⌣}{B}+(\overset{⌣}{\mu }+\overset{⌣}{\vartheta }\alpha^{2})\overset{⌢}{B}}{2(\bar{\mu }l^{2}+\bar{\vartheta })(\overset{⌣}{\mu }+\overset{⌣}{\vartheta }\alpha^{2})l^{2}}+\\ \;\;\;-\frac{\sqrt{{\left[\alpha^{4}l^{2}(\bar{\mu }l^{2}+\bar{\vartheta })\overset{⌣}{B}-(\overset{⌣}{\mu }+\overset{⌣}{\vartheta }\alpha^{2})\overset{⌢}{B}\right]}^{2}+4\alpha^{4}l^{2}(\bar{\mu }l^{2}+\bar{\vartheta })(\overset{⌣}{\mu }+\overset{⌣}{\vartheta }\alpha^{2}){\bar{B}}^{2}}}{2(\bar{\mu }l^{2}+\bar{\vartheta })(\overset{⌣}{\mu }+\overset{⌣}{\vartheta }\alpha^{2})l^{2}},\\ {\left(\omega_{+}\right)}^{2}=\frac{\alpha^{4}l^{2}(\bar{\mu }l^{2}+\bar{\vartheta })\overset{⌣}{B}+(\overset{⌣}{\mu }+\overset{⌣}{\vartheta }\alpha^{2})\overset{⌢}{B}}{2(\bar{\mu }l^{2}+\bar{\vartheta })(\overset{⌣}{\mu }+\overset{⌣}{\vartheta }\alpha^{2})l^{2}}+\\ \;\;\;+\frac{\sqrt{{\left[\alpha^{4}l^{2}(\bar{\mu }l^{2}+\bar{\vartheta })\overset{⌣}{B}-(\overset{⌣}{\mu }+\overset{⌣}{\vartheta }\alpha^{2})\overset{⌢}{B}\right]}^{2}+4\alpha^{4}l^{2}(\bar{\mu }l^{2}+\bar{\vartheta })(\overset{⌣}{\mu }+\overset{⌣}{\vartheta }\alpha^{2}){\bar{B}}^{2}}}{2(\bar{\mu }l^{2}+\bar{\vartheta })(\overset{⌣}{\mu }+\overset{⌣}{\vartheta }\alpha^{2})l^{2}}, \end{array}$$

where ω_−_ is *the lower-order (fundamental) free vibration frequency*, and ω_+_ is *the higher-order free vibration frequency* of the plate band under consideration according to *the tolerance model*.

Proceeding similarly within the asymptotic model, using the Ritz method, expressions for the maximum energies can be written down:

(38) $$U_{\max}^{AM}=\frac{1}{2}(\overset{⌣}{B}A_{W}^{2}\alpha^{4}+2\bar{B}A_{W}A_{Q}\alpha^{2}+\overset{⌢}{B}A_{Q}^{2}),\;\;K_{\max}^{AM}=\frac{1}{2}(\overset{⌣}{\mu }+\overset{⌣}{\vartheta }\alpha^{2})A_{W}^{2}\omega^{2}.$$

Applying conditions (32) to formulae (38) after some manipulations, the following system of linear algebraic equations is obtained:

(39) $$\begin{array}{l} A_{W}[\overset{⌣}{B}\alpha^{4}-(\overset{⌣}{\mu }+\overset{⌣}{\vartheta }\alpha^{2})\omega^{2}]+A_{Q}\bar{B}\alpha^{2}=0,\\ A_{W}\bar{B}\alpha^{2}+A_{Q}\overset{⌢}{B}=0. \end{array}$$

Similarly, as above for the tolerance model, the determinant of the system (39) should be assigned to zero, obtaining the characteristic equation for the plate band under consideration according to the asymptotic model:

(40) $$-\overset{⌢}{B}(\overset{⌣}{\mu }+\overset{⌣}{\vartheta }\alpha^{2})\omega^{2}+\overset{⌣}{B}\overset{⌢}{B}\alpha^{4}-{\left(\bar{B}\alpha^{2}\right)}^{2}=0.$$

The solution to Equation (40) takes the form:

(41) $$\omega^{2}=\frac{\overset{⌣}{B}\overset{⌢}{B}-{\bar{B}}^{2}}{(\overset{⌣}{\mu }+\overset{⌣}{\vartheta }\alpha^{2})\overset{⌢}{B}}\alpha^{4},$$

which is *the lower-order (fundamental) free vibration frequency* ω of the tolerance-periodic plate band according to *the asymptotic model*.

The above analytical results make it possible to observe that the influence of microstructure size can be studied in the tolerance model in terms of higher-order free vibration frequencies, (37)_2_, while only lower-order free vibration frequencies can be analysed in the asymptotic model, (41).

### 4.3. Calculation of Free Vibration Frequencies

Dimensionless frequency parameters are introduced:

(42) $$\Omega_{-}^{2}=\frac{12(1-\nu^{2})\rho^{\prime }}{E^{\prime }}L^{2}{\left(\omega_{-}\right)}^{2},\;\;\Omega_{+}^{2}=\frac{12(1-\nu^{2})\rho^{\prime }}{E^{\prime }}L^{2}{\left(\omega_{+}\right)}^{2};\;\;\Omega^{2}=\frac{12(1-\nu^{2})\rho^{\prime }}{E^{\prime }}L^{2}\omega^{2},$$

with the free vibration frequencies ω_−_, ω_+_, and ω determined by Equations (37) and (41), respectively.

**Figure 3:**
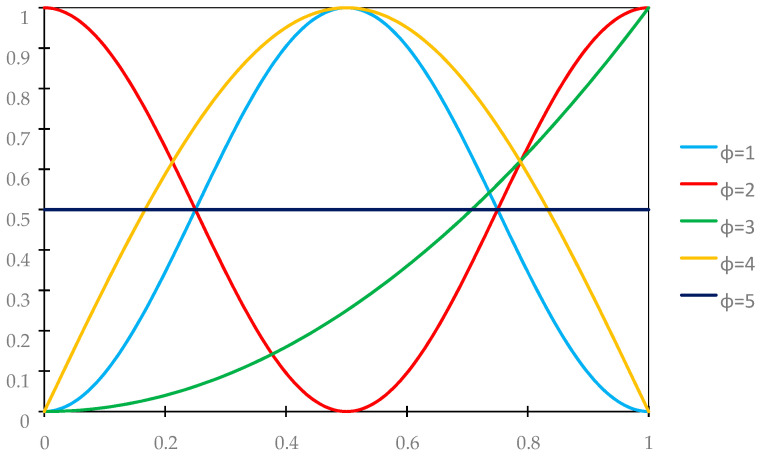
Graphs of distribution functions of properties according to (43)–(47).

The analysis will be carried out for five distribution functions of material properties γ(*x*), whose periodic approximations are assumed as follows, cf. Figure 3:

- variant 1 (ϕ = 1)

(43) $$\tilde{\gamma }(x)=\sin^{2}(\pi x/L);$$

- variant 2 (ϕ = 2)

(44) $$\tilde{\gamma }(x)=\cos^{2}(\pi x/L);$$

- variant 3 (ϕ = 3)

(45) $$\tilde{\gamma }(x)={\left(x/L\right)}^{2};$$

- variant 4 (ϕ = 4)

(46) $$\tilde{\gamma }(x)=\sin(\pi x/L);$$

- variant 5 (ϕ = 5)

(47) $$\tilde{\gamma }(x)=0.5.$$

The results of the free vibration frequency calculations of the tolerance-periodic plate bands shown in the graphs in Figure 4, Figure 5, Figure 6, Figure 7, Figure 8, Figure 9, Figure 10, Figure 11, Figure 12, Figure 13, Figure 14, Figure 15, Figure 16, Figure 17, Figure 18 and Figure 19 were obtained using formulas (42) and (37), (41).

Calculations are made for different types of support (cf. (23)–(26)) and for various distribution functions of properties (formulae (43)–(47)).

The Poisson’s ratio of ν = 0.3 is assumed in the calculations; the thickness of the considered plate band satisfies the condition *d*/*l* = 0.1. The wave number α corresponds to the first form of natural vibration of the homogeneous plate band for each considered support case, i.e., α = π for (23), α = 4.7300 for (24), α = 3.9266 for (25), and α = 1.8751 for (26).

**Figure 4:**
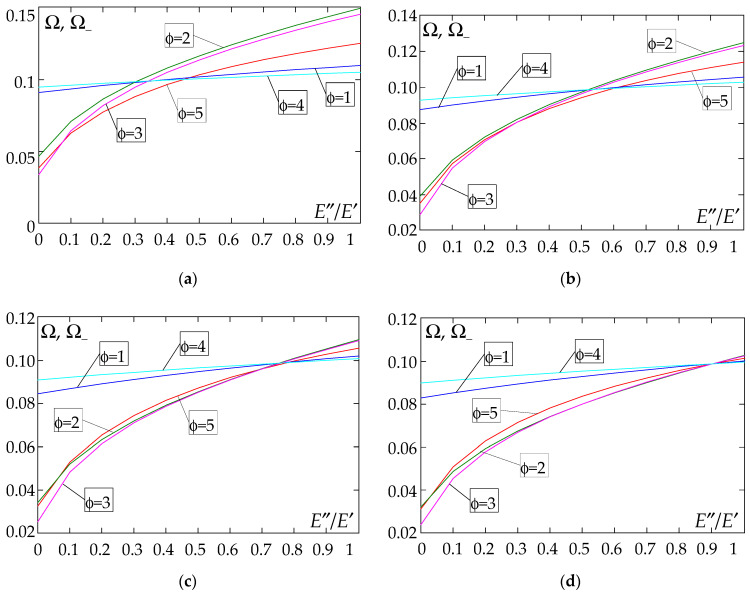
Plot of the lower frequency parameters Ω, Ω_ for a simply supported plate band as a function of the parameter *E*″/*E*′ for a fixed ratio: (**a**) ρ″/ρ′ = 0.25; (**b**) ρ″/ρ′ = 0.50; (**c**) ρ″/ρ′ = 0.75; (**d**) ρ″/ρ′ = 0.90.

**Figure 5:**
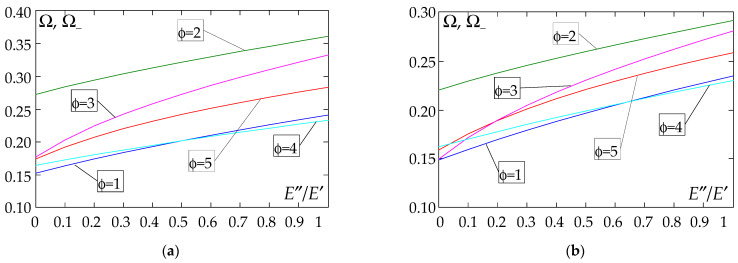

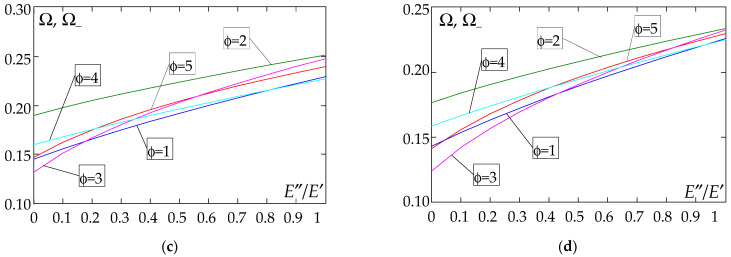
Plot of the lower frequency parameters Ω, Ω_ for a clamped plate band as a function of the parameter *E*″/*E*′ for a fixed ratio: (**a**) ρ″/ρ′ = 0.25; (**b**) ρ″/ρ′ = 0.50; (**c**) ρ″/ρ′ = 0.75; (**d**) ρ″/ρ′ = 0.90.

**Figure 6:**
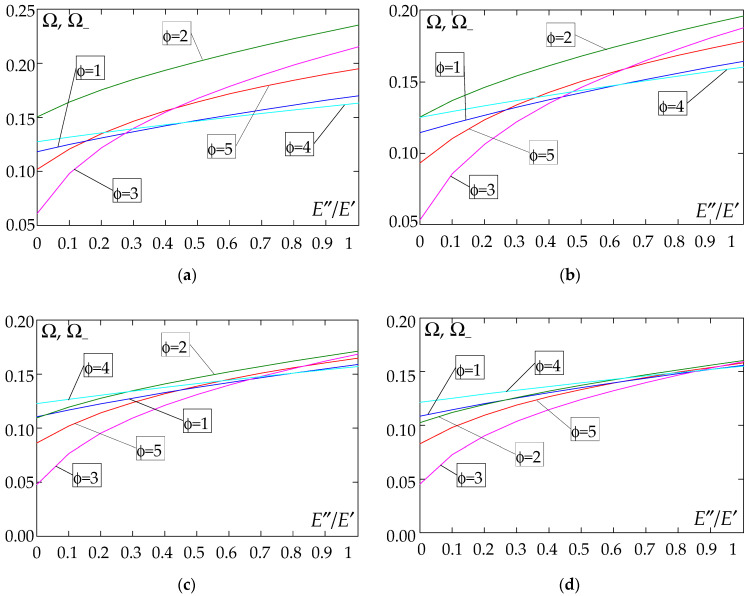
Plot of the lower frequency parameters Ω, Ω_ for a clamped–hinged plate band as a function of the parameter *E*″/*E*′ for a fixed ratio: (**a**) ρ″/ρ′ = 0.25; (**b**) ρ″/ρ′ = 0.50; (**c**) ρ″/ρ′ = 0.75; (**d**) ρ″/ρ′ = 0.90.

**Figure 7:**
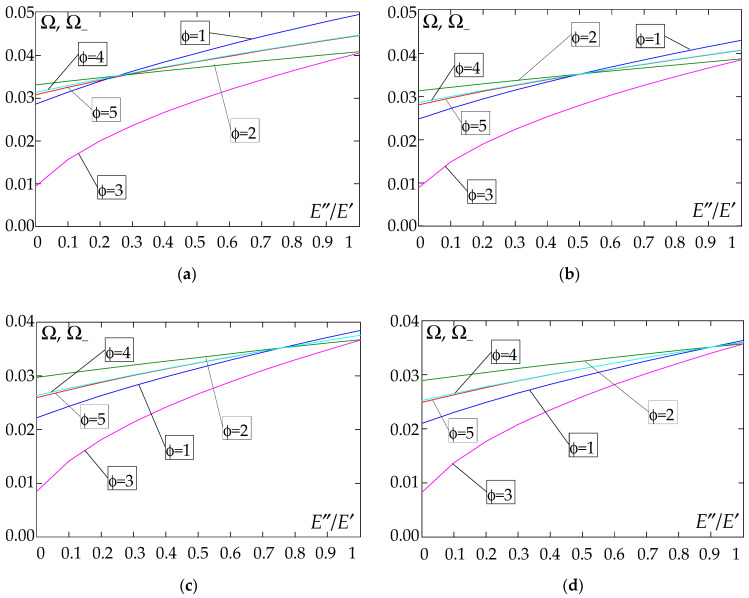
Plot of the lower frequency parameters Ω, Ω_ for a cantilever plate band as a function of the parameter *E*″/*E*′ for a fixed ratio: (**a**) ρ″/ρ′ = 0.25; (**b**) ρ″/ρ′ = 0.50; (**c**) ρ″/ρ′ = 0.75; (**d**) ρ″/ρ′ = 0.90.

**Figure 8:**
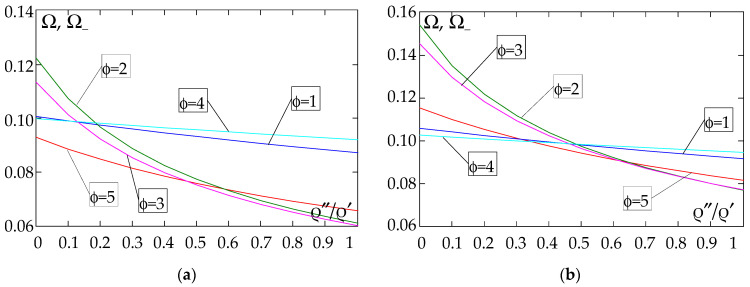

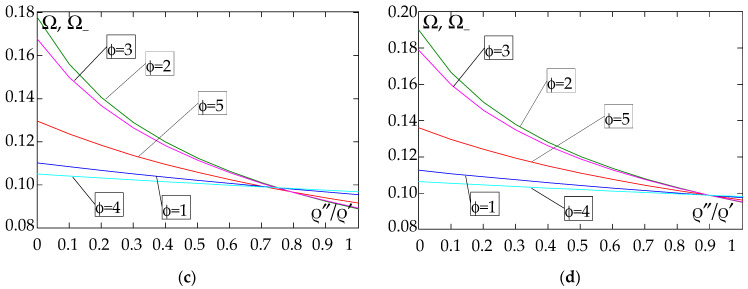
Plot of the lower frequency parameters Ω, Ω_ for a simply supported plate band as a function of the parameter ρ″/ρ′ for a fixed ratio: (**a**) *E*″/*E*′ = 0.25; (**b**) *E*″/*E*′ = 0.50; (**c**) *E*″/*E*′ = 0.75; (**d**) *E*″/*E*′ = 0.90.

**Figure 9:**
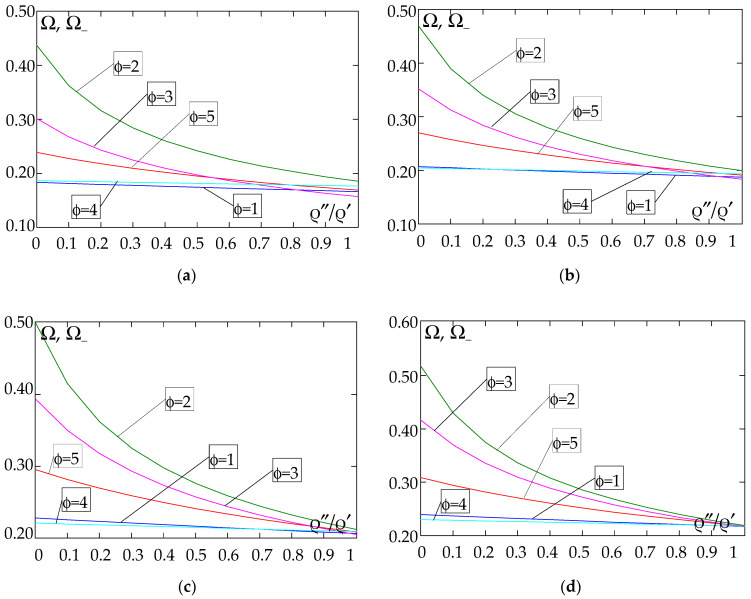
Plot of the lower frequency parameters Ω, Ω_ for a clamped plate band as a function of the parameter ρ″/ρ′ for a fixed ratio: (**a**) *E*″/*E*′ = 0.25; (**b**) *E*″/*E*′ = 0.50; (**c**) *E*″/*E*′ = 0.75; (**d**) *E*″/*E*′ = 0.90.

**Figure 10:**
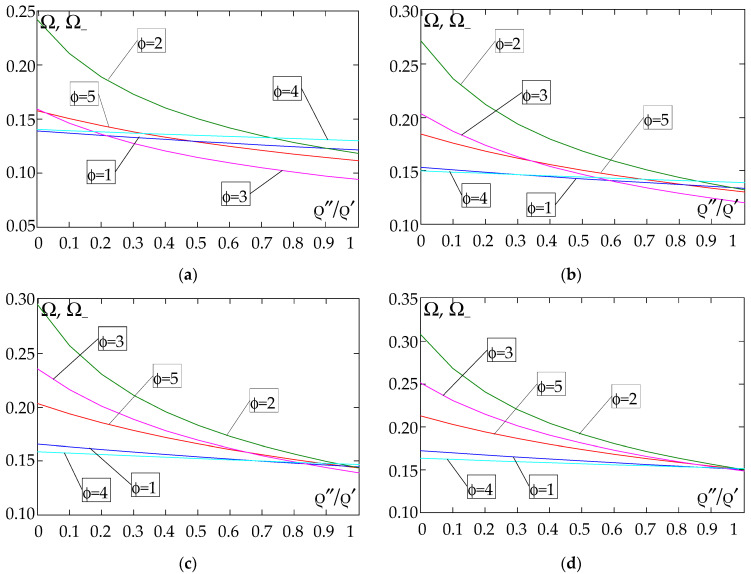
Plot of the lower frequency parameters Ω, Ω_ for a clamped–hinged plate band as a function of the parameter ρ″/ρ′ for a fixed ratio: (**a**) *E*″/*E*′ = 0.25; (**b**) *E*″/*E*′ = 0.50; (**c**) *E*″/*E*′ = 0.75; (**d**) *E*″/*E*′ = 0.90.

**Figure 11:**
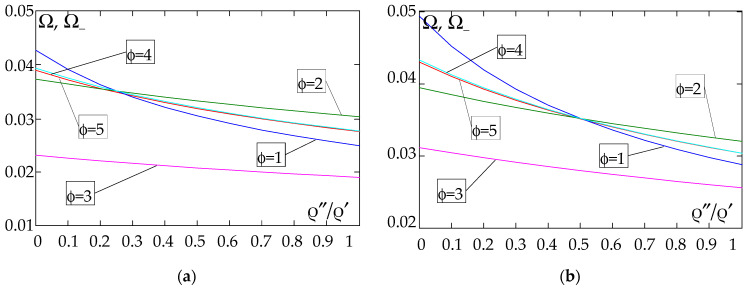

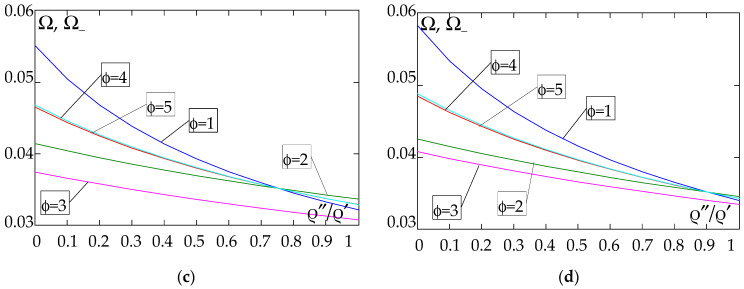
Plot of the lower frequency parameters Ω, Ω_ for a cantilever plate band as a function of the parameter ρ″/ρ′ for a fixed ratio: (**a**) *E*″/*E*′ = 0.25; (**b**) *E*″/*E*′ = 0.50; (**c**) *E*″/*E*′ = 0.75; (**d**) *E*″/*E*′ = 0.90.

Figure 4, Figure 5, Figure 6 and Figure 7 show the dependence of the lower frequency parameters Ω, Ω_ on the Young’s modulus quotient *E*″/*E*′ (for ρ″/ρ′ = 0.25, ρ″/ρ′ = 0.50, ρ″/ρ′ = 0.75, ρ″/ρ′ = 0.90). Figure 8, Figure 9, Figure 10 and Figure 11 present the curves of the lower frequency parameters Ω, Ω_ as a function of the density quotient ρ″/ρ′ (for *E*″/*E*′ = 0.25, *E*″/*E*′ = 0.50, *E*″/*E*′ = 0.75, *E*″/*E*′ = 0.90). The individual curves correspond to assumed distribution functions of the properties, cf. (43)–(47).

Analysing the graphs presented in Figure 4, Figure 5, Figure 6, Figure 7, Figure 8, Figure 9, Figure 10 and Figure 11, the following observations can be made:

(1) The values of the lower frequencies determined according to the tolerance model and the asymptotic model are almost identical.

(2) The following can be observed:

- For a simply supported plate band, the smoothest increase (Figure 4) or decrease (Figure 8) in the values of the lower frequencies is for the distribution function of properties $\tilde{\gamma }(x)=\sin(\pi x/L)$, ϕ = 4; the same applies to a clamped band (Figure 5 and Figure 9, respectively) and a clamped–hinged plate band (Figure 6 and Figure 10, respectively).

- For a cantilever plate band, the smoothest increase (Figure 7) in the values of Ω, Ω_ is for the distribution function of properties $\tilde{\gamma }(x)=\cos^{2}(\pi x/L)$, ϕ = 2, while the decrease (Figure 11) in these values is for the distribution function of properties $\tilde{\gamma }(x)={\left(x/L\right)}^{2}$, ϕ = 3.

(3) The highest values of the lower frequency parameters Ω, Ω_ at fixed parameters *d*/*l* and ρ″/ρ′ are obtained for the following:

- A simply supported plate band for the distribution function of properties $\tilde{\gamma }(x)=\sin(\pi x/L)$, ϕ = 4, when $E^{\prime \prime }/E^{\prime }<{E^{\prime \prime }}_{0}/{E^{\prime }}_{0}$, and for the function $\tilde{\gamma }(x)=\cos^{2}(\pi x/L)$, ϕ = 2, when $E^{\prime \prime }/E^{\prime }>{E^{\prime \prime }}_{0}/{E^{\prime }}_{0}$, Figure 4.

- A plate band clamped on both edges for the distribution function of properties $\tilde{\gamma }(x)=\cos^{2}(\pi x/L)$, ϕ = 2, when $E^{\prime \prime }/E^{\prime }\in [0,1]$, Figure 5.

- A clamped–hinged plate band:

o when ρ″/ρ′ = 0.25 and ρ″/ρ′ = 0.50—for the distribution function of properties $\tilde{\gamma }(x)=\cos^{2}(\pi x/L)$, ϕ = 2, when $E^{\prime \prime }/E^{\prime }\in [0,1]$, Figure 6.

o when ρ″/ρ′ = 0.75 and ρ″/ρ′ = 0.90—for the distribution function of properties $\tilde{\gamma }(x)=\sin(\pi x/L)$, ϕ = 4, when $E^{\prime \prime }/E^{\prime }<{E^{\prime \prime }}_{0}/{E^{\prime }}_{0}$, and for the function $\tilde{\gamma }(x)=\cos^{2}(\pi x/L)$, ϕ = 2, when $E^{\prime \prime }/E^{\prime }>{E^{\prime \prime }}_{0}/{E^{\prime }}_{0}$, Figure 6.

- A cantilever plate band for the distribution function of properties $\tilde{\gamma }(x)=\cos^{2}(\pi x/L)$, ϕ = 2, when $E^{\prime \prime }/E^{\prime }<{E^{\prime \prime }}_{0}/{E^{\prime }}_{0}$, and for the function $\tilde{\gamma }(x)=\sin^{2}(\pi x/L)$, ϕ = 1, when $E^{\prime \prime }/E^{\prime }>{E^{\prime \prime }}_{0}/{E^{\prime }}_{0}$, Figure 7.

(4) The lowest values of the lower frequency parameters Ω, Ω_ at fixed parameters *d*/*l* and ρ″/ρ′ are obtained for the following:

- A simply supported plate band for the distribution function of properties $\tilde{\gamma }(x)={\left(x/L\right)}^{2}$, ϕ = 3, when $E^{\prime \prime }/E^{\prime }<{E^{\prime \prime }}_{0}/{E^{\prime }}_{0}$, and for the function $\tilde{\gamma }(x)=\sin(\pi x/L)$, ϕ = 4, when $E^{\prime \prime }/E^{\prime }>{E^{\prime \prime }}_{0}/{E^{\prime }}_{0}$, Figure 4; the same applies to a clamped–hinged plate band, Figure 6.

- A clamped plate band:

o when ρ″/ρ′ = 0.25 and ρ″/ρ′ = 0.50—for the distribution function of properties $\tilde{\gamma }(x)=\sin^{2}(\pi x/L)$, ϕ = 1, when $E^{\prime \prime }/E^{\prime }<{E^{\prime \prime }}_{0}/{E^{\prime }}_{0}$, and for the function $\tilde{\gamma }(x)=\sin(\pi x/L)$, ϕ = 4, when $E^{\prime \prime }/E^{\prime }>{E^{\prime \prime }}_{0}/{E^{\prime }}_{0}$, Figure 5.

o when ρ″/ρ′ = 0.75 and ρ″/ρ′ = 0.90—for the distribution function of properties $\tilde{\gamma }(x)={\left(x/L\right)}^{2}$, ϕ = 3, when $E^{\prime \prime }/E^{\prime }<{E^{\prime \prime }}_{0}/{E^{\prime }}_{0}$, and for the function $\tilde{\gamma }(x)=\sin(\pi x/L)$, ϕ = 4, when $E^{\prime \prime }/E^{\prime }>{E^{\prime \prime }}_{0}/{E^{\prime }}_{0}$, Figure 5.

- A cantilever plate band for the distribution function of properties $\tilde{\gamma }(x)={\left(x/L\right)}^{2}$, ϕ = 3, when $E^{\prime \prime }/E^{\prime }\in [0,1]$, Figure 7.

(5) The highest values of the lower frequency parameters Ω, Ω_ at fixed *d*/*l* and *E*″/*E*′ parameters are obtained for the following:

- A simply supported plate band for the distribution function of properties $\tilde{\gamma }(x)=\cos^{2}(\pi x/L)$, ϕ = 2, when $\rho^{\prime \prime }/\rho^{\prime }<{\rho^{\prime \prime }}_{0}/{\rho^{\prime }}_{0}$, and for the function $\tilde{\gamma }(x)=\sin(\pi x/L)$, ϕ = 4, when $\rho^{\prime \prime }/\rho^{\prime }>{\rho^{\prime \prime }}_{0}/{\rho^{\prime }}_{0}$, Figure 8; the same applies to a clamped–hinged plate band, Figure 10.

- A clamped plate band for the distribution function of properties $\tilde{\gamma }(x)=\sin^{2}(\pi x/L)$, ϕ = 1, when $\rho^{\prime \prime }/\rho^{\prime }\in [0,1]$, Figure 9.

- A cantilevered plate band for the distribution function of properties $\tilde{\gamma }(x)=\sin^{2}(\pi x/L)$, ϕ = 1, when $\rho^{\prime \prime }/\rho^{\prime }<{\rho^{\prime \prime }}_{0}/{\rho^{\prime }}_{0}$, and for the function $\tilde{\gamma }(x)=\cos^{2}(\pi x/L)$, ϕ = 2, when $\rho^{\prime \prime }/\rho^{\prime }>{\rho^{\prime \prime }}_{0}/{\rho^{\prime }}_{0}$, Figure 11.

(6) The lowest values of the lower frequency parameters Ω, Ω_ at fixed parameters *d*/*l* and *E*″/*E*′ are obtained for:

- A simply supported plate band:

o when *E*″/*E*′ = 0.25—for the distribution function of properties $\tilde{\gamma }(x)=0.5$, ϕ = 5, when $\rho^{\prime \prime }/\rho^{\prime }<{\rho^{\prime \prime }}_{0}/{\rho^{\prime }}_{0}$, and for the function $\tilde{\gamma }(x)=\sin(\pi x/L)$, ϕ = 4, when $\rho^{\prime \prime }/\rho^{\prime }>{\rho^{\prime \prime }}_{0}/{\rho^{\prime }}_{0}$, Figure 8; the same applies to a clamped–hinged plate band, Figure 10.

o when *E*″/*E*′ = 0.50, *E*″/*E*′ = 0.75, and *E*″/*E*′ = 0.90—for the distribution function of properties $\tilde{\gamma }(x)=\sin(\pi x/L)$, ϕ = 4, when $\rho^{\prime \prime }/\rho^{\prime }\in [0,1]$, Figure 8; the same applies to a clamped–hinged plate band, Figure 10.

- A clamped plate band:

o when *E*″/*E*′ = 0.25—for the distribution function of properties $\tilde{\gamma }(x)=\sin^{2}(\pi x/L)$, ϕ = 1, when $\rho^{\prime \prime }/\rho^{\prime }<{\rho^{\prime \prime }}_{0}/{\rho^{\prime }}_{0}$, and for the function $\tilde{\gamma }(x)={\left(x/L\right)}^{2}$, ϕ = 3, when $\rho^{\prime \prime }/\rho^{\prime }>{\rho^{\prime \prime }}_{0}/{\rho^{\prime }}_{0}$, Figure 9.

o when *E*″/*E*′ = 0.50, *E*″/*E*′ = 0.75, and *E*″/*E*′ = 0.90—for the distribution function of properties $\tilde{\gamma }(x)=\sin(\pi x/L)$, ϕ = 4, when $\rho^{\prime \prime }/\rho^{\prime }<{\rho^{\prime \prime }}_{0}/{\rho^{\prime }}_{0}$, and for the function $\tilde{\gamma }(x)={\left(x/L\right)}^{2}$, ϕ = 3, when $\rho^{\prime \prime }/\rho^{\prime }>{\rho^{\prime \prime }}_{0}/{\rho^{\prime }}_{0}$, Figure 9.

- A cantilever plate band for the distribution function of properties $\tilde{\gamma }(x)={\left(x/L\right)}^{2}$, ϕ = 3, when $\rho^{\prime \prime }/\rho^{\prime }\in [0,1]$, Figure 11.

**Figure 12:**
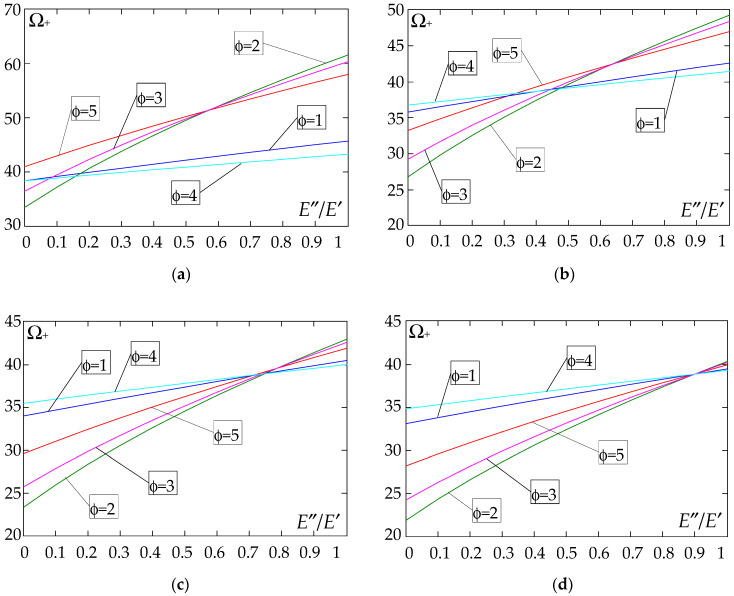
Plot of the higher frequency parameters Ω_+_ for a simply supported plate band as a function of the parameter *E*″/*E*′ for a fixed ratio: (**a**) ρ″/ρ′ = 0.25; (**b**) ρ″/ρ′ = 0.50; (**c**) ρ″/ρ′ = 0.75; (**d**) ρ″/ρ′ = 0.90.

**Figure 13:**
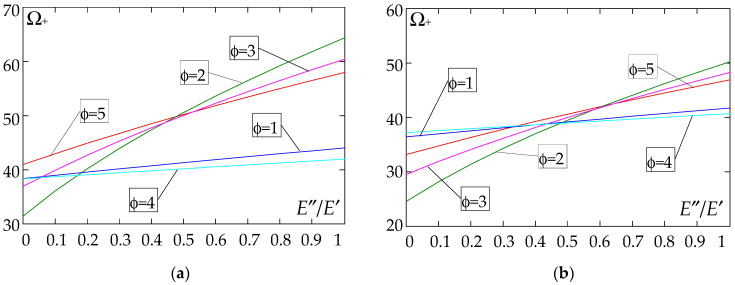

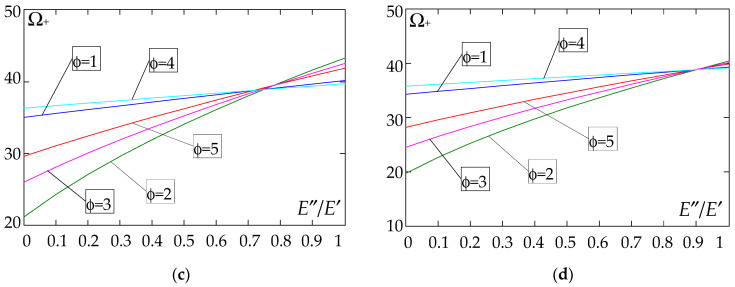
Plot of the higher frequency parameters Ω_+_ for a clamped plate band as a function of the parameter *E*″/*E*′ for a fixed ratio: (**a**) ρ″/ρ′ = 0.25; (**b**) ρ″/ρ′ = 0.50; (**c**) ρ″/ρ′ = 0.75; (**d**) ρ″/ρ′ = 0.90.

**Figure 14:**
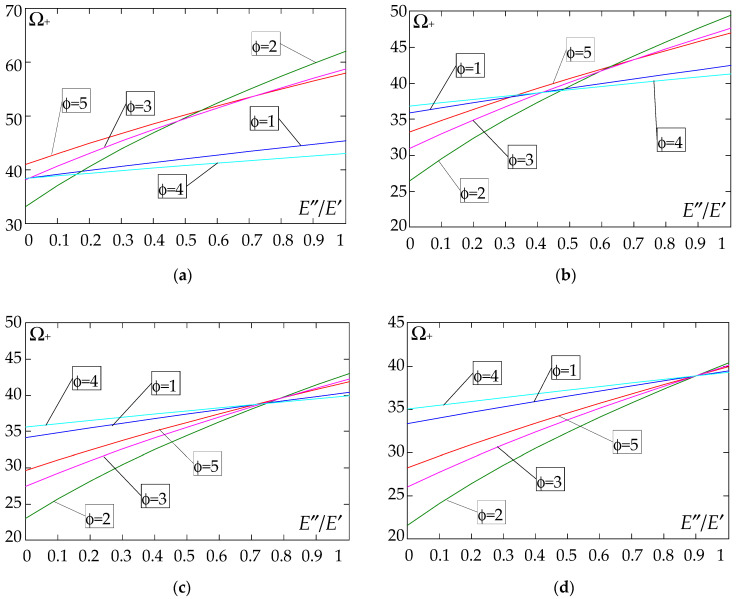
Plot of the higher frequency parameters Ω_+_ for a clamped–hinged plate band as a function of the parameter *E*″/*E*′ for a fixed ratio: (**a**) ρ″/ρ′ = 0.25; (**b**) ρ″/ρ′ = 0.50; (**c**) ρ″/ρ′ = 0.75; (**d**) ρ″/ρ′ = 0.90.

**Figure 15:**
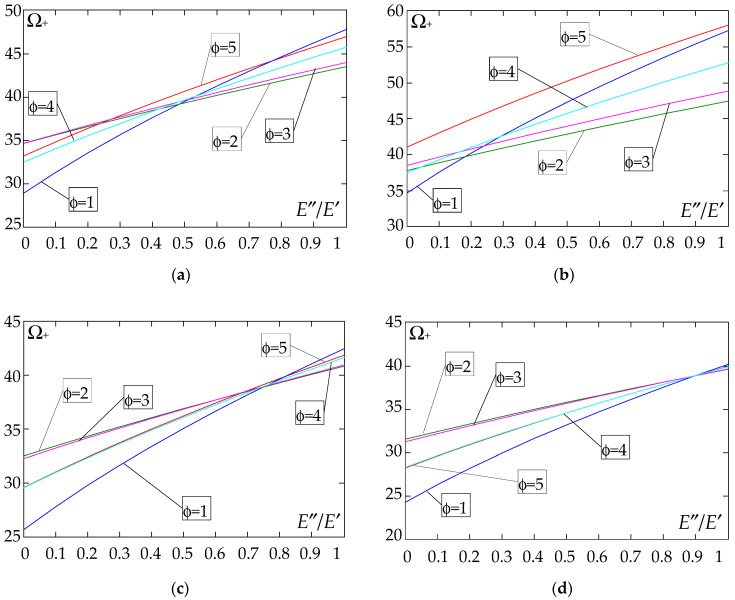
Plot of the higher frequency parameters Ω_+_ for a cantilever plate band as a function of the parameter *E*″/*E*′ for a fixed ratio: (**a**) ρ″/ρ′ = 0.25; (**b**) ρ″/ρ′ = 0.50; (**c**) ρ″/ρ′ = 0.75; (**d**) ρ″/ρ′ = 0.90.

The next graphs in Figure 12, Figure 13, Figure 14 and Figure 15 show the dependence of the higher frequency parameters Ω_+_ on the Young’s modulus quotient parameter *E*″/*E*′ for fixed density quotient ratios.

**Figure 16:**
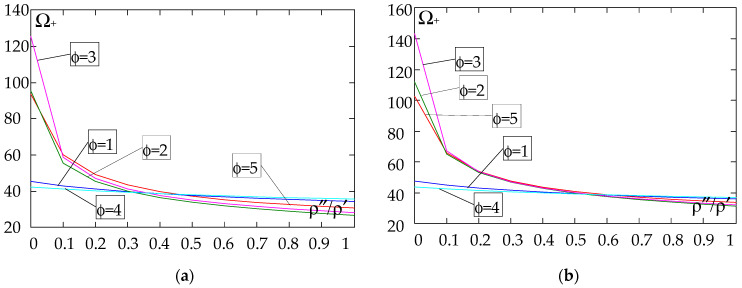

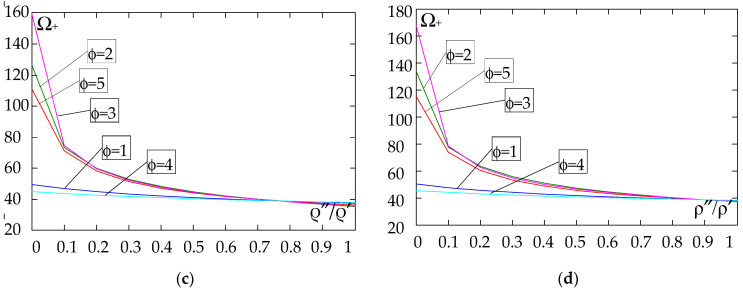
Plot of the higher frequency parameters Ω_+_ for a simply supported plate band as a function of the parameter ρ″/ρ′ for a fixed ratio: (**a**) *E*″/*E*′ = 0.25; (**b**) *E*″/*E*′ = 0.50; (**c**) *E*″/*E*′ = 0.75; (**d**) *E*″/*E*′ = 0.90.

**Figure 17:**
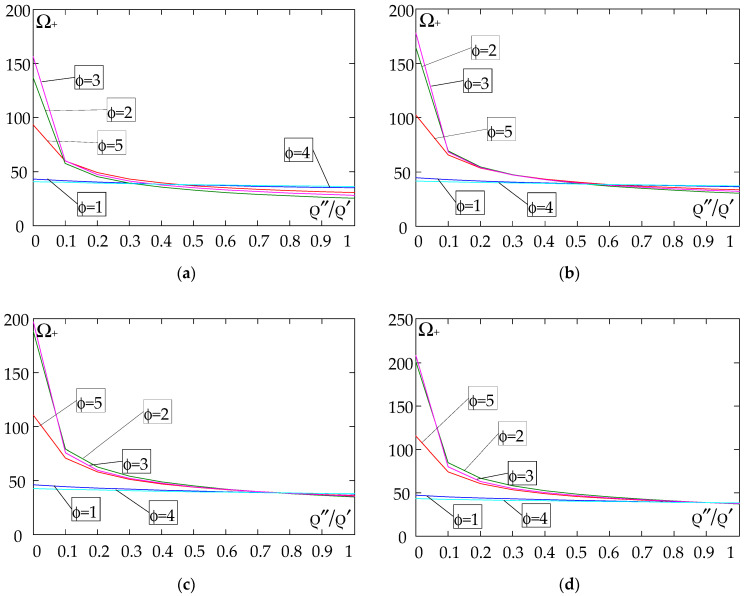
Plot of the higher frequency parameters Ω_+_ for a clamped plate band as a function of the parameter ρ″/ρ′ for a fixed ratio: (**a**) *E*″/*E*′ = 0.25; (**b**) *E*″/*E*′ = 0.50; (**c**) *E*″/*E*′ = 0.75; (**d**) *E*″/*E*′ = 0.90.

**Figure 18:**
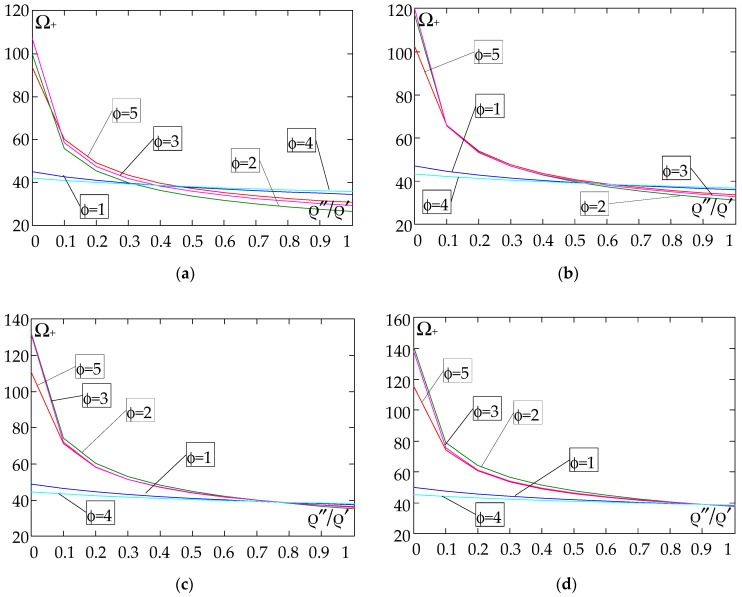
Plot of the higher frequency parameters Ω_+_ for a clamped–hinged plate band as a function of the parameter ρ″/ρ′ for a fixed ratio: (**a**) *E*″/*E*′ = 0.25; (**b**) *E*″/*E*′ = 0.50; (**c**) *E*″/*E*′ = 0.75; (**d**) *E*″/*E*′ = 0.90.

**Figure 19:**
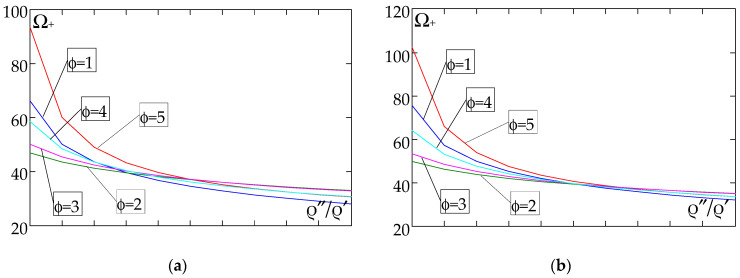

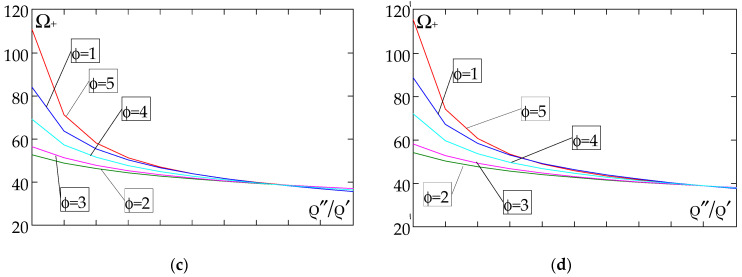
Plot of the higher frequency parameters Ω_+_ for a cantilever plate band as a function of the parameter ρ″/ρ′ for a fixed ratio: (**a**) *E*″/*E*′ = 0.25; (**b**) *E*″/*E*′ = 0.50; (**c**) *E*″/*E*′ = 0.75; (**d**) *E*″/*E*′ = 0.90.

Figure 16, Figure 17, Figure 18 and Figure 19 show graphs of the dependence of the higher frequency parameters Ω_+_ as a function of the density quotient ρ″/ρ′ (for *E*″/*E*′ = 0.25, *E*″/*E*′ = 0.50, *E*″/*E*′ = 0.75, *E*″/*E*′ = 0.90).

Based on Figure 12, Figure 13, Figure 14, Figure 15, Figure 16, Figure 17, Figure 18 and Figure 19, we can make the following observations:

(1) The following can be seen:

- For a simply supported plate band, the smoothest increase (Figure 12) or decrease (Figure 16) in the values of the higher frequency parameters Ω+ is for the distribution function of properties $\tilde{\gamma }(x)=\sin(\pi x/L)$, ϕ = 4; the same applies to a clamped band (Figure 13 and Figure 17, respectively) and a clamped–hinged plate band (Figure 14 and Figure 18, respectively);

- For a cantilever plate band, the smoothest increase (Figure 15) or decrease (Figure 19) in the values of the higher frequency parameters Ω+ is for the distribution function of properties $\tilde{\gamma }(x)=\cos^{2}(\pi x/L)$, ϕ = 2.

(2) The highest values of the higher frequency parameters Ω+ at fixed *d*/*l* and ρ″/ρ′ are obtained for the following:

- A simply supported plate band:

o when ρ″/ρ′ = 0.25—for the distribution function of properties $\tilde{\gamma }(x)=0.5$, ϕ = 5, when $E^{\prime \prime }/E^{\prime }<{E^{\prime \prime }}_{0}/{E^{\prime }}_{0}$, and for the function $\tilde{\gamma }(x)=\cos^{2}(\pi x/L)$, ϕ = 2, when $E^{\prime \prime }/E^{\prime }>{E^{\prime \prime }}_{0}/{E^{\prime }}_{0}$, Figure 12; the same applies to a clamped band, Figure 13, and a clamped–hinged plate band, Figure 14.

o when ρ″/ρ′ = 0.50, ρ″/ρ′ = 0.75, and ρ″/ρ′ = 0.90—for the distribution function of properties $\tilde{\gamma }(x)=\sin(\pi x/L)$, ϕ = 4, when $E^{\prime \prime }/E^{\prime }<{E^{\prime \prime }}_{0}/{E^{\prime }}_{0}$, and for the function $\tilde{\gamma }(x)=\cos^{2}(\pi x/L)$, ϕ = 2, when $E^{\prime \prime }/E^{\prime }>{E^{\prime \prime }}_{0}/{E^{\prime }}_{0}$, Figure 12; the same applies to a clamped band, Figure 13, and a clamped–hinged plate band, Figure 14.

- A cantilever plate band:

o when ρ″/ρ′ = 0.25—for the distribution function of properties $\tilde{\gamma }(x)=0.5$, ϕ = 5, when $E^{\prime \prime }/E^{\prime }\in [0,1]$, Figure 15.

o when ρ″/ρ′ = 0.50, ρ″/ρ′ = 0.75, and ρ″/ρ′ = 0.90—for the distribution function of properties $\tilde{\gamma }(x)=\cos^{2}(\pi x/L)$, ϕ = 2, when $E^{\prime \prime }/E^{\prime }<{E^{\prime \prime }}_{0}/{E^{\prime }}_{0}$, and for the function $\tilde{\gamma }(x)=\sin^{2}(\pi x/L)$, ϕ = 1, when $E^{\prime \prime }/E^{\prime }>{E^{\prime \prime }}_{0}/{E^{\prime }}_{0}$, Figure 15.

(3) The lowest values of higher frequency parameters Ω+ at fixed *d*/*l* and ρ″/ρ′ are obtained for the following:

- A simply supported plate band for the distribution function of properties $\tilde{\gamma }(x)=\cos^{2}(\pi x/L)$, ϕ = 2, when $E^{\prime \prime }/E^{\prime }<{E^{\prime \prime }}_{0}/{E^{\prime }}_{0}$, and for the function $\tilde{\gamma }(x)=\sin(\pi x/L)$, ϕ = 4, when $E^{\prime \prime }/E^{\prime }>{E^{\prime \prime }}_{0}/{E^{\prime }}_{0}$, Figure 12; the same applies to a clamped band, Figure 13, and a clamped–hinged plate band, Figure 14.

- A cantilever plate band for the distribution function of properties $\tilde{\gamma }(x)=\sin^{2}(\pi x/L)$, ϕ = 1, when $E^{\prime \prime }/E^{\prime }<{E^{\prime \prime }}_{0}/{E^{\prime }}_{0}$, and for the function $\tilde{\gamma }(x)=\cos^{2}(\pi x/L)$, ϕ = 2, when $E^{\prime \prime }/E^{\prime }>{E^{\prime \prime }}_{0}/{E^{\prime }}_{0}$, Figure 15.

(4) The highest values of higher frequency parameters Ω+ at fixed *d*/*l* and *E*″/*E*′ are obtained for:

- A simply supported plate band for the distribution function of properties $\tilde{\gamma }(x)={\left(x/L\right)}^{2}$, ϕ = 3, when $\rho^{\prime \prime }/\rho^{\prime }<{\rho^{\prime \prime }}_{0}/{\rho^{\prime }}_{0}$, and for the function $\tilde{\gamma }(x)=\sin(\pi x/L)$, ϕ = 4, when $\rho^{\prime \prime }/\rho^{\prime }>{\rho^{\prime \prime }}_{0}/{\rho^{\prime }}_{0}$, Figure 16; the same applies to a clamped band, Figure 17, and a clamped–hinged plate band, Figure 18;

- A cantilever plate band for the distribution function of properties $\tilde{\gamma }(x)=0.5$, ϕ = 5, when $\rho^{\prime \prime }/\rho^{\prime }<{\rho^{\prime \prime }}_{0}/{\rho^{\prime }}_{0}$, and for the function $\tilde{\gamma }(x)={\left(x/L\right)}^{2}$, ϕ = 3, when $\rho^{\prime \prime }/\rho^{\prime }>{\rho^{\prime \prime }}_{0}/{\rho^{\prime }}_{0}$, Figure 19.

(5) The lowest values of the higher frequency parameters Ω+ at fixed *d*/*l* and *E*″/*E*′ are obtained for the following:

- A simply supported plate band for the distribution function of properties $\tilde{\gamma }(x)=\sin(\pi x/L)$, ϕ = 4, when $\rho^{\prime \prime }/\rho^{\prime }<{\rho^{\prime \prime }}_{0}/{\rho^{\prime }}_{0}$, and for the function $\tilde{\gamma }(x)=\cos^{2}(\pi x/L)$, ϕ = 2, when $\rho^{\prime \prime }/\rho^{\prime }>{\rho^{\prime \prime }}_{0}/{\rho^{\prime }}_{0}$, Figure 16; the same applies to a clamped band, Figure 17, and a clamped–hinged plate band, Figure 18.

- A cantilever plate band for the distribution function of properties $\tilde{\gamma }(x)=\cos^{2}(\pi x/L)$, ϕ = 2, when $\rho^{\prime \prime }/\rho^{\prime }<{\rho^{\prime \prime }}_{0}/{\rho^{\prime }}_{0}$, and for the function $\tilde{\gamma }(x)=\sin^{2}(\pi x/L)$, ϕ = 1, when $\rho^{\prime \prime }/\rho^{\prime }>{\rho^{\prime \prime }}_{0}/{\rho^{\prime }}_{0}$, Figure 19.

## 5. Comparison of Fundamental Frequencies Calculated Using the Tolerance Model (TM) and the Finite Element Method (FEM)

This chapter presents a plate band analysis using the finite element method. The purpose of this analysis is to compare the results obtained with the finite element method and with the tolerance model.

The subject of the calculation is a plate band spanning *L* = *L*_1_ = 10 m along the *x*_1_ axis, simply supported at the edges. For the calculations, the plate band under consideration is assumed to have a width of *L*_2_ = 50 m, along the *x*_2_ axis. The characteristic dimension of the base cell is *l* = 1 m. The plate band thickness is *d* = 10 cm. The considered plate is made of two materials. Material 1 is characterised by the Young’s modulus *E*′ and the mass density ρ′, which correspond to those of steel. Material 2, on the other hand, is an arbitrary isotropic material whose characteristics *E*″ and ρ″ are defined with respect to Material 1. Both materials have the same Poisson’s ratio ν′ = ν″ = ν.

Material characteristic values adopt:

(48) $$\begin{array}{lll} E^{\prime }=210\;\mathrm{Gpa}, & \rho^{\prime }=7860\;\mathrm{kg}/m^{3}, & \nu^{\prime }=0.3\;,\\ \xi =E^{\prime \prime }/E^{\prime }, & \zeta =\rho^{\prime \prime }/\rho^{\prime }, & \nu^{\prime \prime }=0.3\;. \end{array}$$

The following Table 1 shows a comparison of the results of the fundamental lower free vibration frequencies obtained from the tolerance model (Ω_−_) with the free vibration frequencies obtained from Abaqus software v6.14 (Ω_0_) for the distribution functions of the properties: ϕ = 1: $\tilde{\gamma }(x)=\sin^{2}(\pi x/L)$; ϕ = 2: $\tilde{\gamma }(x)=\cos^{2}(\pi x/L)$; ϕ = 5: $\tilde{\gamma }(x)=0.5$.

Abaqus is used for the finite element calculations. The band is modelled with shell elements, four-node S4R. The number of elements generated by the programme is 51,500. A mesh with a mesh size of 0.1 is adopted. Prior to the calculations, a convergence analysis is carried out when compacting the mesh.

The comparisons are made only for the fundamental lower free vibration frequencies, because they can only be calculated in the framework of the commercial computer programs of the finite element method.

The results are shown in the form of dimensionless frequency parameters, defined according to Equation (42)_1_ for the fundamental lower free vibration frequencies by the tolerance model Ω_−_, and for the frequencies obtained from the finite element method given below Ω_0_:

(49) $$\Omega_{0}^{2}=\frac{12(1-\nu^{2})\rho^{\prime }}{E^{\prime }}L^{2}{\left(\omega_{0}\right)}^{2},$$

where the free vibration frequency ω_0_, is determined by the program Abaqus.

The difference parameter between the results according to the finite element method and the tolerance model is defined by the formula:

(50) $$\varepsilon =\left|\frac{\Omega_{0}-\Omega_{\_}}{\Omega_{\_}}\right|100\%.$$

Results are obtained for these tolerance-periodic plate bands for the following ratios: Poisson’s ratios ν′ = ν″ = 0.3; *E*″/*E*′ = 0.3, 0.5, 0.75, 1.0; ρ″/ρ′ = 0.3, 0.5, 0.7, 1.0. Additionally the first lower frequency parameters for the proper homogeneous plate band with these boundary conditions (ν′ = ν″ = 0.3; *E*″/*E*′ = 1; ρ″/ρ′ = 1) are shown, calculated by applying the tolerance model (TM) (Ω_−_ = 0.0299), the classical analytical solution (CPT) (Ω_C_ = 0.0299), and FEM (Ω_0_ = 0.0297).

From the obtained calculation results, the following can be observed:

- The calculation results obtained from the tolerance model (TM) are in agreement with the results obtained from the FEM analysis.

- As the ratio *E*″/*E*′ increases, the relative error between the results obtained according to TM and FEM decreases; an increase in ρ″/ρ′ has no effect on the error considered.

- The largest relative errors are observed in the case of the plate band, for which large differences between Young’s moduli are considered, with the largest differences found for the function ϕ = 1—γ(*x*) = sin^2^(π*x*/*L*)—up to 12%, and the smallest for the function ϕ = 5—γ(*x*) = 0.5—about 4%; for structures with small material disproportions (*E*″/*E*′ ∈ [0.75;1]), the relative error is less than 1.3%; as the stiffness differences (Young’s moduli) of the materials in the cell increase, the differences between the frequency values from the tolerance model and the finite element method also increase.

- In each case considered, the results obtained from the FE analysis are lower than those obtained from the tolerance modelling (TM) procedure.

## 6. Some Final Remarks

The work presented here is concerned with the free vibrations of thin plate bands with different modes of support, with a tolerance-periodic structure at the microlevel and a functionally graded structure in planes parallel to the mid-plane of the plate at the macrolevel. The basis for the considerations is Kirchhoff’s thin plate theory. By considering issues within this theory, equations with strongly oscillating, discontinuous, and tolerance-periodic functional coefficients are obtained. The resulting equations are too difficult to apply directly to the analysis of special cases. Therefore, various averaging methods are used to replace the initial equations with equations with slowly varying coefficients. However, most such methods lead to equations that do not describe the effect of microstructure size.

Using the tolerance modelling technique in this paper, equations with the smooth and slowly varying coefficients of the considered tolerance-periodic plate bands are obtained. Two different models, the tolerance model and the asymptotic model, are compared to show the advantages of the tolerance model.

The considerations presented here make it possible to formulate some general comments common to the applications of the tolerance modelling method and to the analysis of various microheterogeneous structures:

1. For *the tolerance model*, a system of differential equations is obtained in which some components depend on the microstructure parameter *l*. This model makes it possible to take into account *the effect of the microstructure size* on the tolerance-periodic thin plate dynamics problems being solved, such as the “higher order” vibrations, related to the microstructure of the plate.
2. The governing equations of the tolerance model make physical sense if their basic unknowns, i.e., the macrodeflection, *W*, and fluctuation amplitudes, *Q^A^*, *A* = 1, …, *N*, satisfy the *a posteriori* condition, i.e., they are slowly varying functions.
3. In the case of *the asymptotic model*, one differential equation is obtained for the macrodeflection and a system of algebraic equations for the fluctuation amplitudes. These equations do not take into account the effect of microstructure size. Only the basic values of the quantities sought are obtained. It should also be noted that the equations of the asymptotic model can be obtained by applying the appropriate asymptotic modelling procedure, as shown in [3,82] and outlined here in Appendix A, or by omitting components that depend on the microstructure parameter in the equations of the tolerance model.
4. Using *the asymptotic model*, only the lower-order (fundamental) vibrations of the tolerance-periodic plates can be analysed.

The equations derived allow an analysis of the free vibration frequencies of tolerance-periodic FGM-type thin plates. To summarise the application part, the following can be stated:

1. Using the Ritz method of the tolerance model, it is possible to derive formulae for lower-order (fundamental) and higher-order free vibration frequencies for different boundary conditions.
2. A number of calculations were carried out for different boundary conditions and different distribution functions of the properties, and the values obtained made it possible to demonstrate the consistency of the results obtained within the tolerance model and the asymptotic model.
3. The use of the finite element method made it possible to compare the results obtained by applying the proposed models for the fundamental free vibration frequencies of a thin tolerance-periodic plate band with a functional gradation of properties. It can be seen that the relative error of the values obtained is related to the material proportions assumed for the plate band under consideration. The greater the differences between the Young’s moduli for Material 1 and Material 2 assumed for the calculations, the greater the relative error obtained for the determined frequencies. Significant differences in the stiffness of cell fragments may limit the applicability of the tolerance model.
4. Both the models—the tolerance and the asymptotic—allow the consideration of lower free vibration (fundamental) frequencies; but only the tolerance model makes it possible to analyse higher free vibration frequencies, associated with the plate band tolerance-periodic microstructure.
5. The values of the lower free frequencies of the tolerance-periodic plate band are dependent on the boundary conditions, as in the case of homogeneous plates.
6. The values of the higher free vibration frequencies of the tolerance-periodic plate bands being considered also depend on the support conditions, unlike in the case of periodic plate bands, cf. [4].
7. The influences of differences in material and geometrical parameters such as the ratio of Young’s modulus (*E*″/*E*′), the ratio of density (ρ″/ρ′), or thickness-to-plate band span (*d*/*L*) on free vibration frequencies are similar in both lower and higher frequency cases.
8. The effect of the distribution function material properties γ(*x*) on the free vibration frequencies, both lower and higher, is quite difficult to describe and different for both types of frequency.
9. The influence of the distribution function of material properties γ(*x*) on the free vibration frequencies, both lower and higher, is also related to material parameters, i.e., Young’s modulus ratio (*E*″/*E*′) and mass density ratio (ρ″/ρ′). In addition, the influence also depends on the support conditions of the plate.

The rather wide analysis of the free vibration frequencies of tolerance-periodic plate bands presented in this paper makes it possible to note the good utility of the tolerance model when considering this type of issue. In future studies, the proposed tolerance model may be employed to analyse the natural vibrations of rectangular plates with different support conditions and to examine forced vibrations, with the influence of elastic foundation also being taken into account. It is also possible to use this model to optimise the plates of this type. This can be realised by selecting appropriate distribution functions of properties, depending on the boundary conditions. Subsequent articles may also extend the present framework by formulating models of similar plates, but with a plate thickness comparable (of the same order) to the cell length.

## Figures and Tables

**Table 1:** Comparison of the results of the free vibration frequencies obtained within the framework of the tolerance model (Ω_−_) with the free vibration frequencies obtained from the Abaqus program (Ω_0_) for three distribution functions of properties: ϕ = 1 − γ(*x*) = sin^2^(π*x*/*L*); ϕ = 2 − γ(*x*) = cos^2^(π*x*/*L*); ϕ = 5 − γ(*x*) = 0.5.

ϕ		*E*″/*E*′ = 1.00			*E*″/*E*′ = 0.75			*E*″/*E*′ = 0.50			*E*″/*E*′ = 0.30		
	ρ″/ρ′	Ω_−_	Ω_0_	ε [%]	Ω_−_	Ω_0_	ε [%]	Ω_−_	Ω_0_	ε [%]	Ω_−_	Ω_0_	ε [%]
1	0.3	0.0329	0.0327	0.61	0.0318	0.0315	0.94	0.0305	0.0293	3.93	0.0294	0.0261	11.22
	0.5	0.0319	0.0318	0.31	0.0309	0.0305	1.29	0.0296	0.0284	4.05	0.0285	0.0253	11.23
	0.7	0.0311	0.0309	0.64	0.0300	0.0297	1.00	0.0288	0.0276	4.17	0.0277	0.0245	11.55
	1.0	0.0299	0.0297	0.67	0.0289	0.0285	1.38	0.0277	0.0265	4.33	0.0267	0.0235	11.99
2	0.3	0.0433	0.0429	0.92	0.0390	0.0385	1.28	0.0338	0.0327	3.25	0.0285	0.0262	8.07
	0.5	0.0378	0.0375	0.79	0.0340	0.0336	1.18	0.0294	0.0285	3.06	0.0248	0.0228	8.06
	0.7	0.0339	0.0337	0.59	0.0305	0.0302	1.00	0.0264	0.0256	3.03	0.0223	0.0204	8.52
	1.0	0.0299	0.0297	0.67	0.0269	0.0266	1.12	0.0233	0.0225	3.43	0.0196	0.0180	8.16
5	0.3	0.0370	0.0368	0.54	0.0344	0.0341	0.87	0.0306	0.0301	1.63	0.0261	0.0251	3.83
	0.5	0.0345	0.0343	0.58	0.0320	0.0317	0.94	0.0285	0.0280	1.75	0.0243	0.0233	4.12
	0.7	0.0324	0.0322	0.62	0.0301	0.0298	1.00	0.0268	0.0263	1.87	0.0228	0.0219	3.95
	1.0	0.0299	0.0297	0.67	0.0277	0.0275	0.72	0.0247	0.0243	1.62	0.0211	0.0202	4.27

## Data Availability

The original contributions presented in this study are included in the article. Further inquiries can be directed to the corresponding authors.

## Appendix A. The Asymptotic Modelling

This Appendix presents the asymptotic modelling procedure, which was shown in the book [82] in the general form. Here, this procedure is outlined for microheterogenous functionally graded plate bands.

The starting point of this procedure is Equation (2). Using denotations ε ∈ (0, 1] as a parameter, $\Delta_{\varepsilon }\equiv [-\varepsilon l/2,\varepsilon l/2]\times \{0\}$ as an interval, and $\Delta_{\varepsilon }(x)\equiv x+\Delta_{\varepsilon }$, $x\in \bar{\Pi }$ as ε-cell, we define ${\tilde{f}}_{\varepsilon }(x,y)\equiv \tilde{f}(x,y/\varepsilon )$, ${\tilde{f}}_{\varepsilon }(x,\cdot )\in H^{1}(\Delta_{\varepsilon })\subset H^{1}(\Delta )$, $y\in \Delta_{\varepsilon }(x)$, $x\in \bar{\Pi }$, for function $\tilde{f}(x,\cdot )\in H^{1}(\Delta )$, $\forall x\in \bar{\Pi }$. Let $h^{A}(\cdot )$, $h^{A}(\cdot )\in HO_{\delta }^{2}(\Pi ,\Delta )$, *A* = 1, …, *N*, be independent functions, and ${\tilde{h}}^{A}(x,\cdot )$ be their periodic approximations, given by ${\tilde{h}}_{\varepsilon }^{A}(x,y)\equiv {\tilde{h}}^{A}(x,y/\varepsilon ),\;y\in \Delta_{\varepsilon }(x)$, for every $x\in \bar{\Pi }$.

The fundamental assumption of the asymptotic modelling is *the asymptotic decomposition* for the deflection *w*(·,*t*):

(A1) $$w_{\varepsilon }(x,y,t)=W(y,t)+\varepsilon^{2}{\tilde{h}}_{\varepsilon }^{A}(x,y)Q^{A}(y,t),\;\;A=1,\ldots ,N,$$

where $y\in \Delta_{\varepsilon }(x),\;t\in (t_{0},t_{1}),$ and functions *w*, *W*, *Q^A^* are continuous and bounded in $\bar{\Pi }$ with their derivatives. Denote $\hat{\partial }{\tilde{h}}_{\varepsilon }^{A}(x,y)\equiv \varepsilon \partial {\tilde{h}}^{A}(x,\bar{y}){|}_{\bar{y}=y/\varepsilon },$ ${\hat{\partial }}^{2}{\tilde{h}}_{\varepsilon }^{A}(x,y)\equiv \varepsilon^{2}\partial^{2}{\tilde{h}}^{A}(x,\bar{y}){|}_{\bar{y}=y/\varepsilon }.$ Taking into account ε⟶0, since $y\in \Delta_{\varepsilon }(x)$, $x\in \bar{\Pi }$, the formulas of derivatives of the deflection $w_{\varepsilon }$ have the form:

(A2) $$\begin{array}{l} w_{\varepsilon }(x,y,t)=W(x,t)+O(\varepsilon ),\;\partial w_{\varepsilon }(x,y,t)=\partial W(y,t)+O(\varepsilon ),\\ \partial^{2}w_{\varepsilon }(x,y,t)=\partial^{2}W(y,t)+{\hat{\partial }}^{2}{\tilde{h}}_{\varepsilon }^{A}(x,y)Q^{A}(y,t)+O(\varepsilon ). \end{array}$$

From the limit passage ε⟶0, terms *O*(ε) are neglected in the above relations. Then Lagrangians ${\tilde{\Lambda }}_{\varepsilon }=\tilde{\Lambda }(x,y/\varepsilon ,\partial W,\dot{W},W,Q^{A}),$ $y\in \Delta_{\varepsilon }(x),\;x\in \bar{\Pi },\;t\in (t_{0},t_{1}),$ are introduced. In the asymptotic procedure for ε⟶0 functions ${\tilde{\Lambda }}_{\varepsilon }$ of *y*/ε, $y\in \Delta_{\varepsilon }(x)$, tend to the averaged function $\Lambda_{0}$. After some manipulations we arrive at the Lagrangian $\Lambda_{0}$:

(A3) $$\begin{array}{c} \Lambda_{0}=-\frac{1}{2}\{(<b>\partial^{2}W+2<b\partial^{2}h^{B}>Q^{B})\partial^{2}W+<\vartheta >\partial \dot{W}\partial \dot{W}-\\ -<\mu >\dot{W}\dot{W}+<b\partial^{2}h^{A}\partial^{2}h^{B}>Q^{A}Q^{B}\}. \end{array}$$

Applying the principle of the stationary of the functional to (A3), the Euler–Lagrange equations are obtained in the form:

(A4) $$\begin{array}{l} \partial^{2}(B\partial^{2}W+B^{A}Q^{A})+m\ddot{W}-\vartheta \partial^{2}\ddot{W}=0,\\ B^{A}\partial^{2}W+B^{AB}Q^{B}=0. \end{array}$$
